# Cyclic AMP-binding protein Epac1 acts as a metabolic sensor to promote cardiomyocyte lipotoxicity

**DOI:** 10.1038/s41419-021-04113-9

**Published:** 2021-09-01

**Authors:** Marion Laudette, Yannis Sainte-Marie, Grégoire Cousin, Dorian Bergonnier, Ismahane Belhabib, Stéphanie Brun, Karina Formoso, Loubna Laib, Florence Tortosa, Camille Bergoglio, Bertrand Marcheix, Jan Borén, Olivier Lairez, Jérémy Fauconnier, Alexandre Lucas, Jeanne Mialet-Perez, Cédric Moro, Frank Lezoualc’h

**Affiliations:** 1 Institut des Maladies Métaboliques et Cardiovasculaires, Inserm, Université Paul Sabatier, UMR 1297-I2MC, Toulouse, France; 2 Department of Molecular and Clinical Medicine, Wallenberg Laboratory, University of Gothenburg and Sahlgrenska University Hospital, Gothenburg, Sweden; 3 Centre Hospitalier Universitaire de Toulouse Rangueil, Toulouse, France; 4 PHYMEDEXP, Université de Montpellier, CNRS, INSERM, CHRU Montpellier, Montpellier, France

**Keywords:** Mechanisms of disease, Cardiomyopathies

## Abstract

Cyclic adenosine monophosphate (cAMP) is a master regulator of mitochondrial metabolism but its precise mechanism of action yet remains unclear. Here, we found that a dietary saturated fatty acid (FA), palmitate increased intracellular cAMP synthesis through the palmitoylation of soluble adenylyl cyclase in cardiomyocytes. cAMP further induced exchange protein directly activated by cyclic AMP 1 (Epac1) activation, which was upregulated in the myocardium of obese patients. Epac1 enhanced the activity of a key enzyme regulating mitochondrial FA uptake, carnitine palmitoyltransferase 1. Consistently, pharmacological or genetic Epac1 inhibition prevented lipid overload, increased FA oxidation (FAO), and protected against mitochondrial dysfunction in cardiomyocytes. In addition, analysis of Epac1 phosphoproteome led us to identify two key mitochondrial enzymes of the the β-oxidation cycle as targets of Epac1, the long-chain FA acyl-CoA dehydrogenase (ACADL) and the 3-ketoacyl-CoA thiolase (3-KAT). Epac1 formed molecular complexes with the Ca^2+^/calmodulin-dependent protein kinase II (CaMKII), which phosphorylated ACADL and 3-KAT at specific amino acid residues to decrease lipid oxidation. The Epac1-CaMKII axis also interacted with the α subunit of ATP synthase, thereby further impairing mitochondrial energetics. Altogether, these findings indicate that Epac1 disrupts the balance between mitochondrial FA uptake and oxidation leading to lipid accumulation and mitochondrial dysfunction, and ultimately cardiomyocyte death.

## Introduction

The heart has a high energy demand and most of the ATP produced comes from mitochondrial oxidative phosphorylation, from which fatty acid oxidation (FAO) contributes to 70% of the energy supply [1]. In metabolic diseases such as obesity and type 2 diabetes mellitus, accumulation of intramyocardial lipids contributes to cardiac defects termed lipotoxic cardiomyopathy [2]. In this cardiac insult, the metabolic flexibility of the myocardium is compromised as illustrated by an increase in the use of fatty acids (FA) as an energy substrate, and a decrease in carbohydrates utilization [3]. In addition, an imbalance between FA uptake and oxidation results in intracellular accumulation of lipid intermediates thereby causing reactive oxygen species (ROS) generation, mitochondrial dysfunction, and cardiomyocyte death [2]. This metabolic alteration contributes to cardiac alteration [4]. Therefore, impaired myocardial metabolism and mitochondrial dysfunction play a critical role in the pathogenesis of lipotoxic cardiomyopathy. However, how myocardial FA metabolism is disrupted during lipotoxic insults yet remains elusive.

Cyclic adenosine monophosphate (cAMP) is a versatile signaling molecule that regulates a large variety of cellular processes, including cardiac contraction, cellular death, and metabolism [5]. Synthesis of cAMP from adenosine triphosphate (ATP) is generated by membrane-bound adenylyl cyclases (AC) upon activation of G-coupled-protein receptors (GPCR) [6]. The type 10 soluble adenylyl cyclase (sAC) constitutes the second source of cAMP and is activated by HCO_3_^−^ and Ca^2+^ to control various cellular processes [7, 8]. In mitochondria, sAC promotes post-translational regulation of mitochondrial oxidative phosphorylation activity via cAMP-dependent protein kinase A (PKA) phosphorylation to stimulate ATP synthesis [9–11]. Interestingly, previous findings reported that the sympathetic nervous system, which is a key regulator of cardiac function, is overstimulated in the diabetic heart and contributes to impaired cAMP-PKA signaling [12, 13]. However, little is known on the role and mechanisms of cAMP action in the control of myocardial metabolism and lipotoxicity induced by lipid overload.

Although PKA has long been considered as the most active kinase in the mitochondrial matrix and the main effector of intra-mitochondrial cAMP [14], the cAMP downstream target, exchange protein directly activated by cAMP 1 (Epac1) has recently gained a lot of interest for its mitochondrial localization and role [15–17]. Epac1 is a guanine-nucleotide-exchange factor (GEF) that mediates the cAMP-dependent but PKA-independent activation of small G-proteins of the Ras superfamily, Rap1 and Rap2 [18, 19]. Compelling evidence is now accumulating about the formation of Epac1 molecular complexes in distinct cellular sites that influence Epac1 signalling and cellular function [17, 20]. With respect to the mitochondrial compartment, Epac1 regulated the dynamic phosphorylation of dynamin-1-like protein (DRP1), which is crucial for mitochondrial fission/fusion balance [21]. Another study demonstrated that, during cardiac ischemia, mitochondrial Epac1 increased Ca^2+^ uptake and ROS production thereby promoting mitochondrial death signalling such as mitochondrial permeability transition pore (MPTP) opening and cytochrome c release, and ultimately cardiomyocyte death [16]. While it is clear that Epac1 is expressed in mitochondria, further studies are needed to determine mitochondrial Epac1 molecular complexes and biological function.

Here, we report that a diet-dependent signal, palmitate regulates the intracellular synthesis of cAMP to activate Epac1, which in turn promotes lipid accumulation and mitochondrial dysfunction. We unravel an unsuspected molecular mechanism by which cAMP-Epac1 signaling induces metabolic inflexibility and contributes to lipotoxicity in cardiomyocytes.

## Results

### Palmitic acid stimulates mitochondrial sAC to activate cAMP-Epac1 signaling

**Fig. 1:**
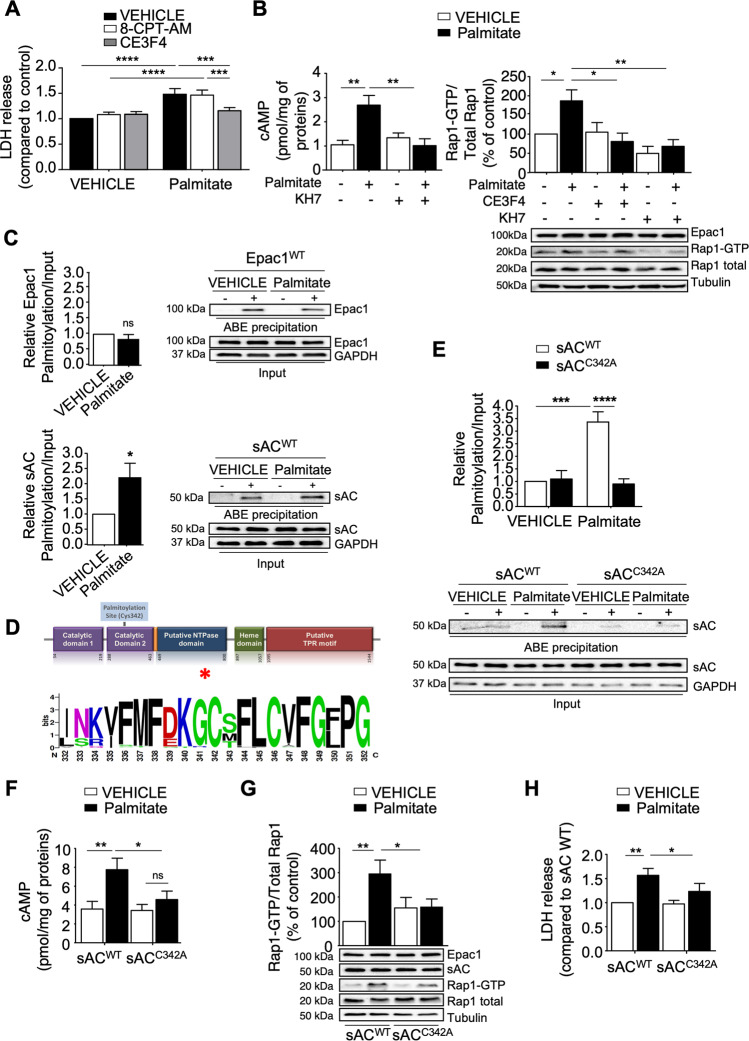
Palmitate treatment increases cAMP production and Epac1 activity through sAC palmitoylation. **A** Measurement of LDH release in cardiomyocytes pretreated with 8-CPT-AM (10 µM, 30 min) or CE3F4 (20 µM, 30 min) and incubated with either palmitate (500 µM, 24 h) or its VEHICLE (BSA-control) (*n* = 6). **B** Intracellular cAMP (*n* = 6) and Rap1-GTP (*n* = 6) levels in cardiomyocytes transfected with Epac1 and pretreated or not with CE3F4 (20 μM, 30 min) or KH7 (20 μM, 30 min) before incubation with palmitate (500 μM, 1 h). **C, E** Quantification of Epac1 (*n* = 5), mitochondrial sAC (sAC^WT^) (*n* = 6) or a mutant sAC^C342A^ palmitoylation in cells transfected with the indicated plasmids and treated with palmitate (500 μM, 1 h) (*n* = 4). **D** Scheme of the functional domains in mammalian sAC and graphical representation with Weblogo software of a multiple sequence alignment of 97 sequences of sAC from different species performed with Consurf software. The mammalian sAC is comprised of two catalytic domains C1 and C2 (violet) localized in the N-terminal domain, followed by an autoinhibitory motif (orange). The C-terminal region contains a putative NTPase domain (blue), an Heme domain (green) and putative TPR modules (red). Within the C2 there is a putative palmitoylation site (C342). **F–H** Intracellular cAMP (**F**, *n* = 6) or Rap1-GTP (**G**, *n* = 6) levels, LDH release (**H**, *n* = 7) in cells transfected with the indicated plasmids. The results are expressed as mean ±SEM and were analysed with a two-way ANOVA/Bonferroni post test. *p < 0.05, **p < 0.01, ***p < 0.001, ****p < 0.0001 vs indicated value. Representative immunoblots are shown.

Palmitate is a major saturated FA in the plasma, and chronic exposure of primary cardiomyocytes to bovine serum albumin (BSA)-bound palmitic acid produces a model of FA-induced lipotoxicity in vitro [22]. Using the LDH and MTT assays, we found that one day treatment with palmitate promoted cardiomyocyte death Epac1 (Fig. 1A, Supplementary Fig. 1). This effect was blocked by a selective Epac1 inhibitor CE3F4 [23], suggesting that palmitate activated Epac1 (Fig. 1A, Supplementary Fig. 1). Accordingly, the Epac1 specific agonist, 8-pCPT-2′-O-Me-cAMP-AM (8-CPT-AM) [24] failed to potentiate the effect of palmitate on cardiomyocyte death (Fig. 1A, Supplementary Fig. 1). Of note, palmitate increased the amount of intracellular cAMP, the cognate activator of Epac1 and thus the active form of Rap1, a direct effector of Epac1 (Fig. 1B and Supplementary Fig. 2A). As expected, a nontoxic FA (Supplementary Fig. 2B), oleate failed to induce Rap1 activation (Supplementary Fig. 2C). Furthermore, a selective sAC inhibitor, KH7, blocked cAMP accumulation and significantly reduced palmitate-induced Epac1 activation in a manner similar to CE3F4 (Fig. 1B). Altogether, these data demonstrate that palmitate stimulates sAC and subsequent cAMP production promotes Epac1 activation, which triggers cardiomyocyte death.

We used a nonradioactive acyl-biotin exchange (ABE) method to determine how palmitate regulates sAC activity [25]. Treatment with a reducing agent, hydroxylamine (“+“) reveals proteins initially palmitoylated and captured by streptavidin before cleavage. The absence of signal in “−“ lane is a negative control corresponding to all initially free cysteines that have been blocked. It confirms that the signal obtained in “+“ lane depends on the activity of hydroxylamine and reflects the state of palmitoylation of the protein. We found that both Epac1 and mitochondrial sAC were palmitoylated under basal condition (Fig. 1C). However, palmitate enhanced sAC palmitoylation but not that of Epac1 (Fig. 1C). Bioinformatic analysis of mitochondrial sAC sequence revealed a potential palmitoylation site on Cys342 of sAC, which is located in the C2 catalytic domain of sAC, close to the ATP and HCO_3_^−^ binding sites [26] and is phylogenetically conserved across sAC orthologs (Fig. 1D and Supplementary Fig. 2D). Remarkably, a mutant sAC with this Cys residue substituted to Ala residue (sAC^C342A^) mutant failed to be palmitoylated compared to sAC^WT^ in palmitate-treated cardiomyocytes (Fig. 1E). This effect of sAC^C342A^ translated on cAMP production and Rap1-GTP (Fig. 1F, G). Accordingly, palmitate-induced cardiomyocyte death was compromised in sAC^C342A^ transfected cells (Fig. 1H). Altogether, these data provide strong evidence that mitochondrial sAC is post-translationally activated by palmitoylation to promote Epac1-induced cardiomyocyte death.

### Epac1 impairs mitochondrial function during a chronic lipotoxic stress

**Fig. 2:**
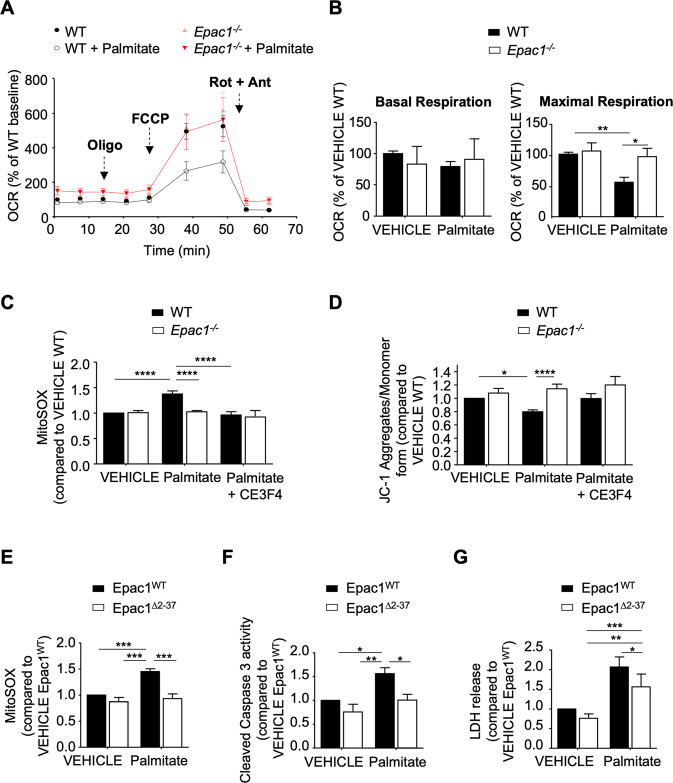
Epac1 promotes mitochondrial aberrations leading to cardiomyocyte death. **A** Measurements of oxygen consumption rate (OCR) in adult cardiomyocytes isolated from WT or *Epac1*^*−/−*^ mice, incubated or not (VEHICLE) with palmitate (150 μM, 2 h) after oligomycin, FCCP, antimycin A or rotenone (1 µM) addition (*n* = 4–5). **B** Graphical representation of basal or maximal respiration of WT and *Epac1*^*−/−*^ cardiomyocytes subjected to palmitate-induced metabolic stress (*n* = 4–5). **C, D** Quantification of mitochondrial ROS using MitoSOX (**C**) and mitochondrial membrane potential (**D**) using JC-1 probe in WT or *Epac1*^*−/−*^ cardiomyocytes pretreated with CE3F4 (20 µM, 30 min) or (VEHICLE) and stimulated with palmitate (150 μM, 2 h) (*n* = 6). **E–G** Primary cardiomyocytes expressing wild-type Epac1 (Epac1^WT^) or mutant deleted for its putative mitochondrial-targeting sequence (Epac1^Δ2–37^) were stimulated with 8-CPT-AM (10 μM, 30 min) and incubated with palmitate (500 μM). Mitochondrial ROS quantification (**E**, *n* = 7), caspase 3 activity (**F**, *n* = 5), or LDH release (**G**, *n* = 11). Results are expressed as mean ±SEM and were analyzed with a two-way ANOVA/Bonferroni post test. *p < 0.05, **p < 0.01, ***p < 0.001, ****p < 0.0001 vs indicated value.

While chronic exposure of palmitate reduced maximal mitochondrial respiration in wild-type (WT) cardiomyocytes, *Epac1*^*−/−*^ cardiomyocytes were resistant to palmitate-induced oxygen consumption rate (OCR) decrease (Fig. 2A, B). In addition, palmitate provoked an increase in mitochondrial ROS (mROS) content assayed with the superoxide sensitive probe MitoSOX Red; superoxide production remained low and similar to control condition in cells devoid of *Epac1*, or in WT cardiomyocytes treated with CE3F4 (Fig. 2C). Consistently, Epac1 genetic or pharmacological inhibition prevented palmitate-induced loss of mitochondrial membrane potential (ΔΨm) in isolated cardiomyocytes (Fig. 2D). Direct activation of Epac1 with 8-CPT-AM had similar effect of palmitate on mitochondrial dysfunction (Supplementary Fig. 3A, B) and cardiomyocyte apoptosis (Supplementary Fig. 3C). Of note, Epac1 pharmacological inhibitors and palmitate failed to dysregulate the expression level of oxidative phosphorylation enzyme complexes indicating that these compounds did not induce lesions of the oxidative phosphorylation system in our experimental conditions (Supplementary Fig. 4). Finally, cardiomyocytes transfected with a mutant form of Epac1 deleted for its putative mitochondrial-targeting sequence (Epac1^∆2–37^, [16]) exhibited less mROS and caspase 3 activation compared to those transfected with the Epac1^WT^ and treated with palmitate (Fig. 2E, F). As expected, palmitate-induced cell death was reduced in Epac1^∆2–37^ expressing cardiomyocytes (Fig. 2G). Together, these data strongly suggest that mitochondrial expression of Epac1 promotes mitochondrial dysfunction and cardiomyocyte death during persistent lipid overload.

### Epac1 promotes mitochondrial FA uptake

**Fig. 3:**
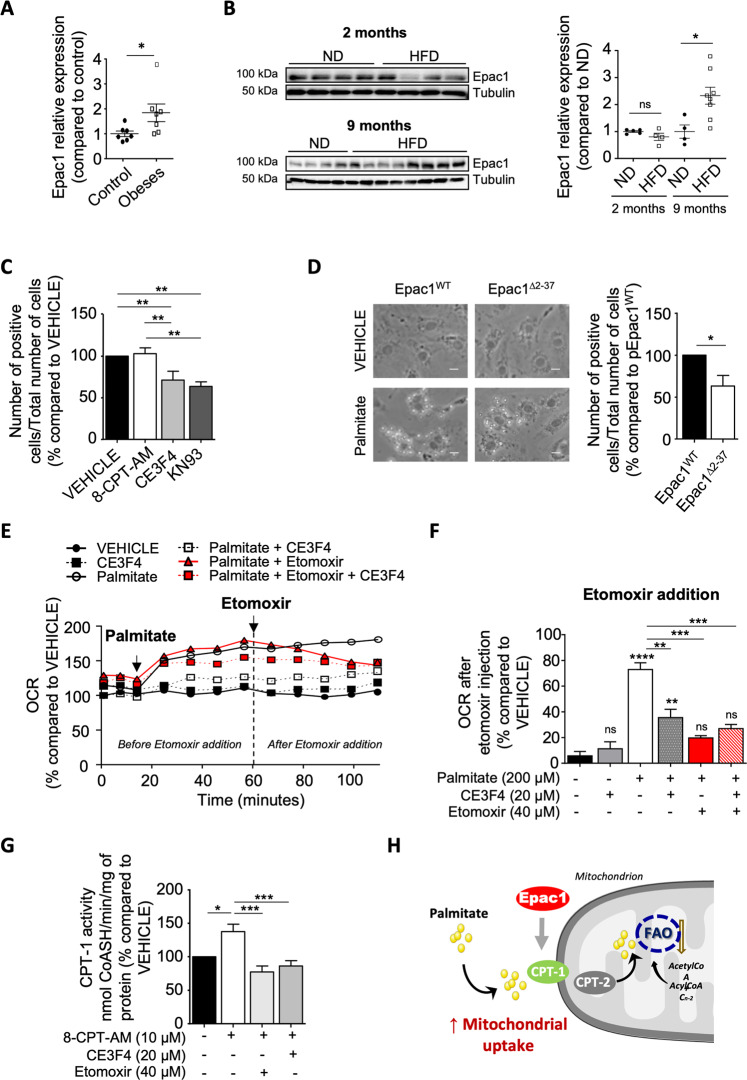
Epac1 promotes lipid accumulation and regulates mitochondrial FA uptake. **A, B** Representative immunoblot and quantification of Epac1 protein in human right atrial appendage tissues from non-obese control or obese patients (*n* = 7), and in cardiac samples from C57Bl6 mice subjected to normal diet (ND) or high-fat diet (HFD) for 2 or 9 months (*n* = 5–8). GAPDH, Tubulin, loading control. **C, D** Quantification of lipid accumulation (**C**) in primary cardiomyocytes pretreated or not (VEHICLE) with 8-CPT-AM (10 μM, 30 min), CE3F4 (20 μM, 30 min), or KN-93 (5 μM, 30 min) and incubated with palmitate (500 μM, 24 h) (*n* = 4–6 different experiments) Scale, 10 μm. In **D** cells were transfected with the indicated plasmids for 24 h and then incubated with palmitate (500 μM, 24 h) (*n* = 5 different experiments). Representatives images are shown on the left panel. **E, F** Measurement of OCR in cardiomyocytes pretreated with CE3F4 (20 μM, 30 min) or not (VEHICLE) after acute treatment of palmitate (500 µM, 15 min) and CPT-1 inhibitor, etomoxir (40 μM, 60 min) (*n* = 2–4). In **F** the graphical representation is shown (*n* = 2–4). **G** CPT-1 activity in cardiomyocytes pretreated or not with 8-CPT-AM (10 μM, 30 min) and then treated with CE3F4 (20 μM, 30 min) or etomoxir (40 µM, 30 min) (*n* = 6–8). **H** Schematic representation of the regulation of CPT-1 activity by Epac1. The results are expressed as mean ±SEM and were analyzed with a two-way ANOVA/Bonferroni post test. In **A**, a Mann–Whitney test was performed. *p < 0.05, **p < 0.01, ***p < 0.001, ****p < 0.0001 vs indicated value. ns not significant.

Obesity increases circulating levels of free FAs that accumulate in the myocardium to promote cardiomyocyte lipotoxicity [2]. Epac1 expression level was upregulated in the myocardium of obese patients compared with that of controls (Fig. 3A and Supplementary Fig. 5A). Similarly, cardiac samples from mice subjected to a high-fat diet (HFD, 60% lipid) for a long term period of 9 months had increased Epac1 expression. Interestingly, Epac1 inhibition with CE3F4 reduced lipid overload in primary cardiomyocytes incubated with palmitate (Fig. 3C and Supplementary Fig. 5B). Because CaMKII was reported to act as a downstream effector of Epac1 in various biological processes [17, 20], we investigated the effect of a CaMKII pharmacological inhibitor, KN-93 on lipid overload. As expected, KN-93 mimicked the effect of CE3F4 on lipid accumulation (Fig. 3C and Supplementary Fig. 5B). The Epac1 agonist, 8-CPT-AM failed to potentiate the effect of palmitate (Fig. 3C) because this FA behaved as a stimulator of Epac1 (Fig. 1B). Interestingly, Epac1^WT^ transfected cells displayed increased lipid accumulation than those overexpressing the mutant Epac1^∆2–37^ that excluded Epac1 from the mitochondria [16] suggesting that cytosolic Epac1 regulates lipid accumulation and/or mitochondrial Epac1 controls FA metabolism (Fig. 3D).

Long-chain FAs are transported across the mitochondrial membrane by the carnitine palmitoyltransferase 1 (CPT-1) [27]. Thus, CPT-1 plays a critical role in mitochondrial FA uptake and constitutes a rate-limiting enzyme in FA oxidation. We therefore addressed whether Epac1 would regulate FA transport inside mitochondria via CPT-1 activity. To test this hypothesis, we measured mitochondrial OCR after a short-term treatment with palmitate and in the presence or absence of a CPT-1 inhibitor, etomoxir (Fig. 3E). As previously described [28], acute stimulation with palmitate provoked an increase in OCR in primary cardiomyocytes, an effect that was inhibited by etomoxir (Fig. 3E, F). Similarly, pretreatment of cells with CE3F4 prevented palmitate-induced augmentation in mitochondrial respiration (Fig. 3E, F). In agreement with these results, 8-CPT-AM significantly increased CPT-1 activity, which was blocked by either etomoxir or CE3F4 (Fig. 3G). Altogether, these results demonstrate that Epac1 increases CPT-1 activity to promote FA uptake inside mitochondria (Fig. 3H).

### Epac1 inhibits metabolic flexibility of cardiomyocytes

**Fig. 4:**
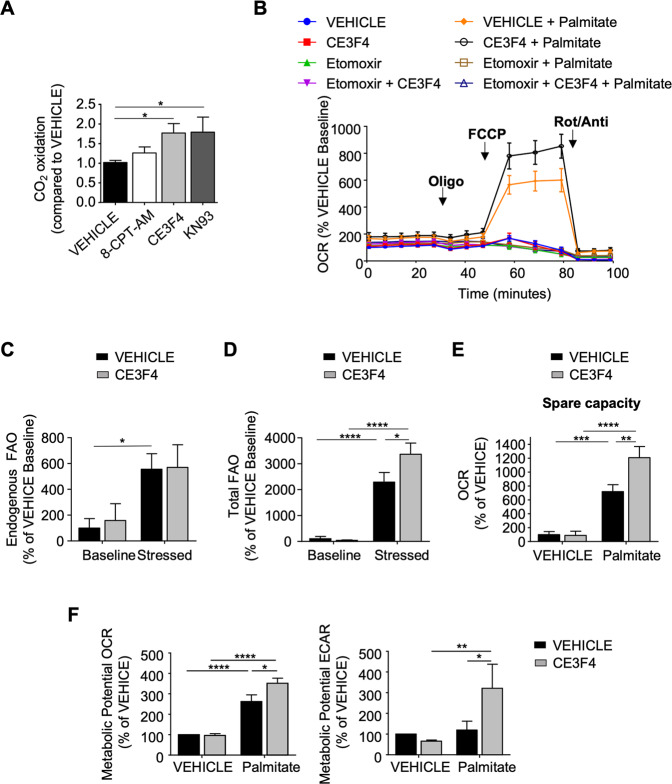
Epac1 regulates FA and carbohydrates oxidation. **A** Determination of FAO in cardiomyocytes by measuring radiolabeled CO_2_ produced by palmitate^14^C. Cells were pretreated or not (VEHICLE) with 8-CPT-AM (10 μM, 30 min), CE3F4 (20 μM, 30 min), or KN-93 (5 μM, 30 min) before addition of palmitate^14^C for 3 h (*n* = 9). **B** Measurement of OCR in cardiomyocytes pretreated with CE3F4 (20 μM, 30 min) or not (VEHICLE) after oligomycin (2 µM), FCCP (2 µM), antimycin A (4 µM), and rotenone (2 µM) addition (*n* = 7). Cells were pretreated with etomoxir (40 μM, 15 min) before palmitate addition (500 μM) at time 0. **C, D** Quantification of endogenous (**C**) or **D** total (endogenous + exogenous) oxidation of FA in baseline and in FCCP-induced stress conditions. **E** Graphical representation of respiratory capacity of cardiomyocytes pretreated with CE3F4 (20 μM, 30 min) or not (VEHICLE) and treated with palmitate (500 µM). **F** Metabolic Potential calculated by the Seahorse XF Cell Energy Phenotype Test Report Generator (Percentage of stressed (FCCP addition) OCR or ECAR over baseline OCR or ECAR. This parameter is the measure of cells’ ability to meet an energy demand via respiration and glycolysis. The results are expressed as mean ±SEM and were analyzed with a two-way ANOVA/Bonferroni post test. *p < 0.05, **p < 0.01, ***p < 0.001, ****p < 0.0001 vs indicated value.

Since mitochondrial FAO capacity is coupled with FA mitochondria uptake [29], we next assessed whether Epac1 would influence palmitate oxidation by measuring the degree of CO_2_ produced from the degradation of radiolabelled [^14^C]-palmitate in cardiomyocytes. Cells treated with either CE3F4 or KN-93 had increased amount of radiolabelled CO_2_ indicating an inhibitory effect of Epac1/CaMKII axis on FAO (Fig. 4A). As expected, direct Epac1 activation with 8-CPT-AM failed to influence FAO because of the stimulating effect of palmitate on Epac1 activation (Fig. 1B). FAO levels were similar in vehicle and CE3F4 treated cardiomyocytes suggesting that Epac1 was not involved in FA metabolism in basal condition (Fig. 4B, C; Supplementary Fig. 6A). On the contrary, pharmacological inhibition of Epac1 with CE3F4 significantly increased FAO levels in palmitate-treated cardiomyocytes (Fig. 4B, D). We used FCCP to measure the ability of cells to respond to increased energy demand (leading to a rapid oxidation of palmitate in this context) and found that CE3F4 enhanced maximal respiration as well as respiratory capacity in FCCP-induced stress conditions compared to untreated cells. These data demonstrate Epac1 inhibition allows cardiomyocytes to better cope with increased energy supply linked to an excess of FA (Fig. 4B, E; Supplementary Fig. 6B).

Next, we sought to determine whether Epac1 also played a role in glucose oxidation. We measured the ExtraCellular Acidification Rate (ECAR) of cardiomyocytes using Seahorse XF24 flow analyzer in the presence of glucose, oligomycin (ATP synthase inhibitor to enhance glycolysis), or 2-deoxyglucose (glycolysis inhibitor). After addition of a saturating concentration of glucose, 8-CPT-AM tended to decrease glycolysis while CE3F4 increased ECAR indicating that Epac1 inhibition enhanced the glycolytic capacity of cardiomyocytes (Supplementary Fig. 6C, D). Of particular importance, metabolic potential measurements using Seahorse Cell Energy Phenotype Test (to determine basal and maximal mitochondrial respiration and glycolytic activity in living cells) showed that palmitate significantly increased the oxidative metabolic potential (OCR) of cardiomyocytes without affecting their glycolytic metabolic potential (ECAR) (Fig. 4E, F). Strikingly, cells treated with CE3F4 exhibited a higher oxidative and glycolytic metabolic potential indicating that Epac1 inhibition prevented FAO-induced glucose oxidation inhibition in FA overload condition (Fig. 4E, F). Altogether, these results show that Epac1 inhibition allows a better metabolic flexibility of cardiomyocytes in the context of excess lipid flux.

### Epac1 targets β-oxidation promoting enzymes through a CaMKII-dependent pathway

**Fig. 5:**
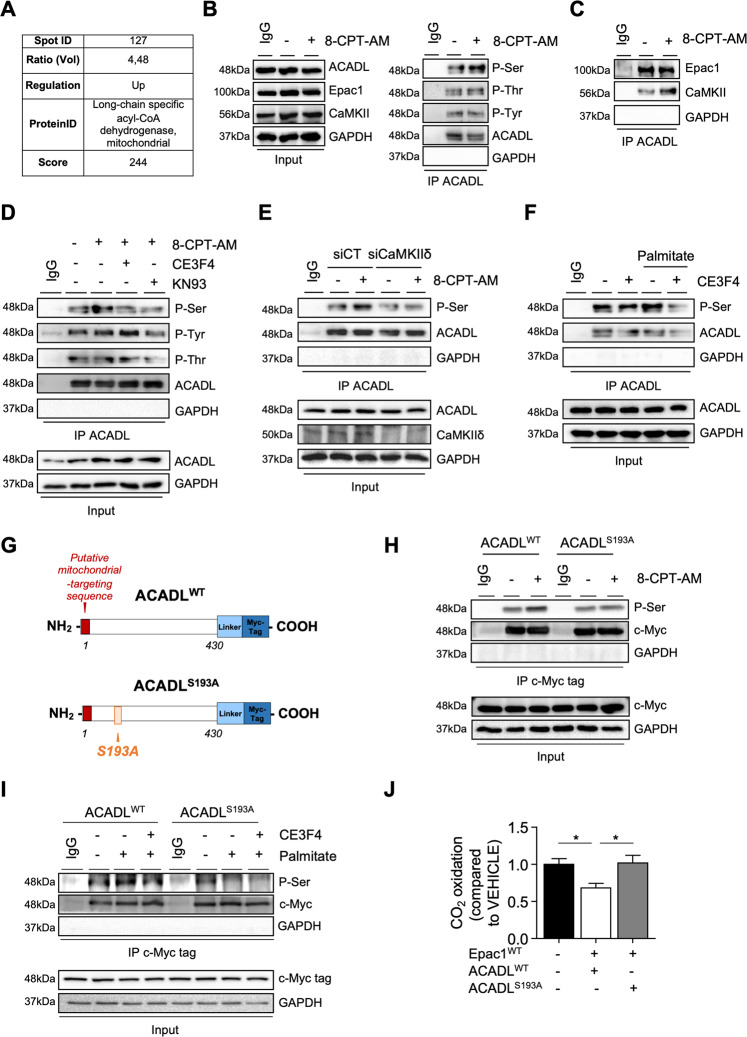
Epac1-CaMKII targets Acyl-CoA dehydrogenase (ACADL) to decrease FA breakdown. **A** Phosphoproteome results. The ratio of volume value is indicated in the table. The change in the intensity of the protein spot (127) is indicated as increased (up) in the stimulated 8-CPT-AM (10 µM, 10 min) vs. control. ID identity. **B** Representative immunoblots showing ACADL phosphorylation at Ser, Thr, and Tyr residues in cardiomyocytes stimulated or not with 8-CPT-AM (10 µM, 30 min). **C** Interaction of ACADL and **D** its phosphoresidues regulation by Epac1 and CaMKII. Cardiomyocytes were preincubated or not with either CE3F4 (20 µM, 30 min) or KN-93 (5 µM, 30 min) and were stimulated or not with 8-CPT-AM (10 µM, 30 min). **E** ACADL-dependent serine phosphorylation after CaMKIIδ silencing (siCaMKIIδ) or not (siCT) following 8-CPT-AM stimulation (10 μM, 30 min). **F** Regulation of ACADL phosphorylation by palmitate (500 μM, 30 min). **G** Schematic representation of ACADL wild type (ACADL^WT^) and mutant form (ACADL^S193A^). **H, I** Phosphorylation of ACADL in cells transfected with the indicated plasmids and stimulated with the indicated compounds. IgG and GAPDH were used as control for immunoprecipitation (IP). Input is a control of cell lysates. The immunoblots are representative of three independent experiments. **J** Determination of FAO by measuring radiolabeled CO_2_ produced by palmitate^14^C in cardiomyocytes expressing wild-type Epac1 (Epac1^WT^) and transfected with either ACADL^WT^ or ACADL^S193A^ (*n* = 14–17) The results are expressed as mean ±SEM and were analyzed with a one-way ANOVA / Bonferroni post test. *p < 0.05.

To further elucidate the molecular mechanisms whereby Epac1 influenced energy metabolism, we next searched for molecular targets of Epac1. We analyzed data from Epac1 phosphoproteome that we performed using two-dimensional electrophoresis coupled to tandem mass spectrometry [16]. Among the phosphopeptides identified, spot 127 and spot 155 corresponded to two mitochondrial enzymes, the long-chain fatty acid acyl-CoA dehydrogenase (ACADL) and the 3-ketoacyl-CoA thiolase (3-KAT) (Fig. 5A, Supplementary Fig. 7A). ACADL and 3-KAT catalyze the first and last key steps in mitochondrial FAO, respectively [1]. In line with the Epac1 phosphoproteome analysis, co-immunoprecipitation experiments showed that Epac1 activation increased ACADL and 3-KAT serine-specific phosphorylation (Fig. 5B and Supplementary Fig. 7B). Furthermore, mitochondrial Epac1, CaMKII, and ACADL or 3-KAT were involved in the same macromolecular complex (Fig. 5C and Supplementary Fig. 5B). Pharmacological inhibition of Epac1 (CE3F4) or CaMKII (KN-93) prevented Epac1-mediated ACADL or 3-KAT phosphorylation (Fig. 5D and Supplementary Fig. 7C).

Interestingly, a bioinformatic analysis of ACADL sequence identified a potential phosphorylation site on Ser193 for CaMKIIδ isoform. Knockdown of CaMKIIδ isoform with specific siRNA (SiCaMKIIδ) blocked 8-CPT-AM-induced ACADL phosphorylation, demonstrating that CaMKIIδ specifically targets ACADL in response to Epac1 activation (Fig. 5E). Of note, palmitate promoted ACADL phosphorylation was prevented by CE3F4 suggesting that palmitate via the sAC-cAMP-Epac1 axis regulates the phosphorylation state of ACADL (Fig. 5F). Transfection of a mutant ACADL with Ser193 substituted to Ala residue (ACADL^S193A^) blocked 8-CPT-AM or palmitate mediated ACADL serine phosphorylation compared to ACADL^WT^ (Fig. 5H, I). Importantly, cells expressing ACADL^S193A^ were resistant to Epac1-induced FAO reduction, as shown by the increased amounts of radiolabelled CO_2_ compared to cells expressing ACADL^WT^ (Fig. 5J). Altogether, these data indicate that Epac1-CaMKII axis targets enzymes of the FAO to decrease FA breakdown through the β-oxidation spiral.

### Epac1 regulates ATP synthase activity

**Fig. 6:**
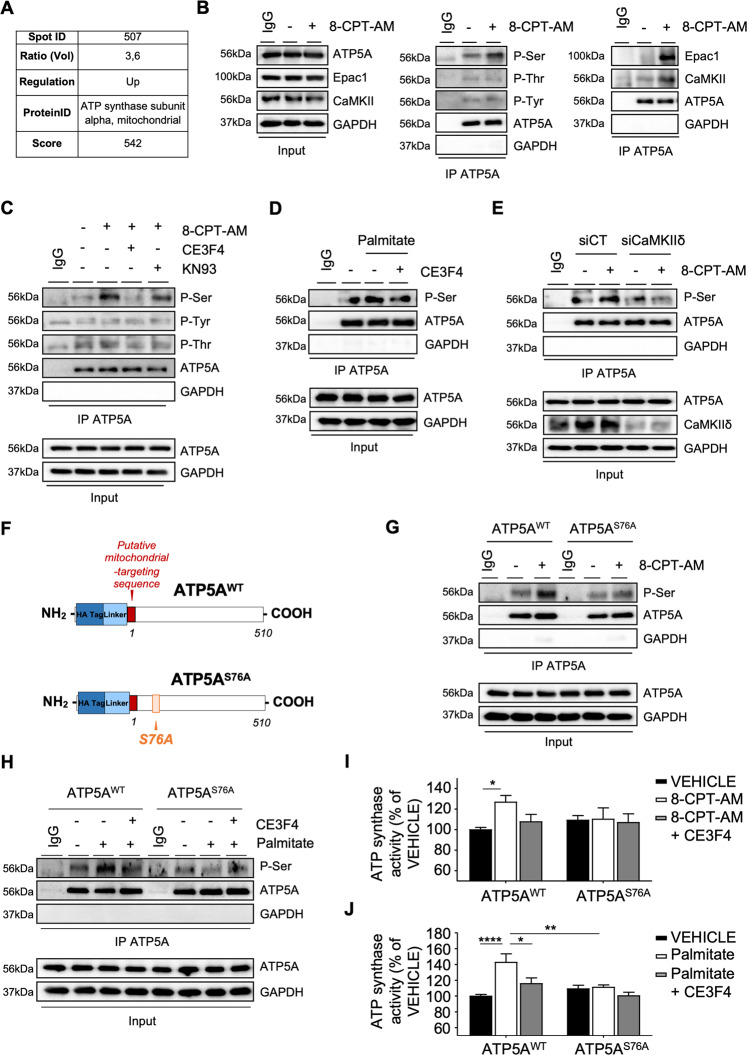
Epac1 phosphorylates the α subunit of ATP synthase through CaMKII and regulates its activity. **A** Phosphoproteome results. The ratio of volume value is indicated in the table. The change in the intensity of the protein spot (507) is indicated as increased (up) in the stimulated 8-CPT-AM (10 µM, 10 min) vs. control. ID, identity. **B** Representative immunoblots showing the interaction of ATP5A with Epac1 or CaMKII, or ATP5A phosphorylation level in cardiomyocytes stimulated or not with 8-CPT-AM (10 µM, 30 min). **C, D** ATP5A phosphorylation in cells preincubated or not with either CE3F4 (20 µM, 30 min) or KN-93 (5 µM, 30 min) and stimulated or not with either 8-CPT-AM (10 µM, 30 min) or palmitate (500 μM, 30 min). **E** ATP5A-dependent serine phosphorylation after CaMKIIδ silencing (siCaMKIIδ) or not (siCT) following 8-CPT-AM stimulation (10 μM, 30 min). **F** Schematic representation of ATP5A wild type (ATP5A^WT^) and mutant form (ATP5A^S76A^). **G, H** Phosphorylation of ATP5A^WT^ or ATP5A^S76A^ in cells pretreated or with CE3F4 (20 μM, 30 min) and stimulated or not with 8-CPT-AM (10 μM, 30 min) or palmitate (500 μM, 30 min). The immunoblots are representative of three independent experiments. IgG and GAPDH were used as control for IP. Input is a control of cell lysates. **I, J** ATP synthase activity in cardiomyocytes transfected with the indicated plasmids and pretreated as in (**G, H**) (*n* = 6). The results are expressed as mean ±SEM and were analyzed with a two-way ANOVA/Bonferroni post test. *p < 0.05, **p < 0.01, ****p < 0.0001 vs control group or indicated value.

The reducing equivalents (NADH and FADH) resulting from the FAO deliver electrons through the distinct complexes of the electron transport chain (ETC) to build up a proton gradient used in fine by the ATP synthase to generate ATP from ADP [1]. Interestingly, the α subunit of ATP synthase (ATP5A) was revealed in Epac1 phosphoproteome (Fig. 6A). Epac1 activation increased ATP5A, Epac1, and CaMKII complex formation as well as ATP5A Ser-specific phosphorylation (Fig. 6B). Pharmacological inhibition of Epac1 or CaMKII with CE3F4 or KN-93 blocked 8-CPT-AM—increased ATP5A Ser phosphorylation level (Fig. 6C). Similarly, palmitate-induced ATP5A phosphorylation was prevented by CE3F4 indicating that palmitate regulates the phosphorylation state of ATP5A via Epac1 (Fig. 6D).

A bioinformatic analysis of the ATP5A sequence identified a potential phosphorylation site on Ser76 for CaMKIIδ isoform. As expected, transfection of SiCaMKIIδ or a mutated form of ATP5 on Ser76 (ATP5A^S76A^) prevented Epac1-mediated ATP5A serine phosphorylation (Fig. 6E, F, G). The ATP5A^S76A^ mutant was also effective in blocking the effect of palmitate on the phosphorylation level of ATP5A^WT^ like CE3F4 (Fig. 6H). This effect also translated on ATP synthase activity (Fig. 6I, J). Finally, we assessed ATP synthase activity in WT and Epac1^−/−^ hearts subjected to chronic HFD. In-gel activity analysis showed that Epac1 genetic inhibition preserved a higher complex V activity and oligomerization after 15 weeks of HFD compared to WT mice (Supplementary Fig. 8) suggesting that Epac1 inhibition allows to maintain ATP production during chronic metabolic stress. Altogether, these data showed that Epac1-CaMKII axis is involved in the regulation of ATP synthase activity leading to an impaired mitochondrial energetics.

## Discussion

**Fig. 7:**
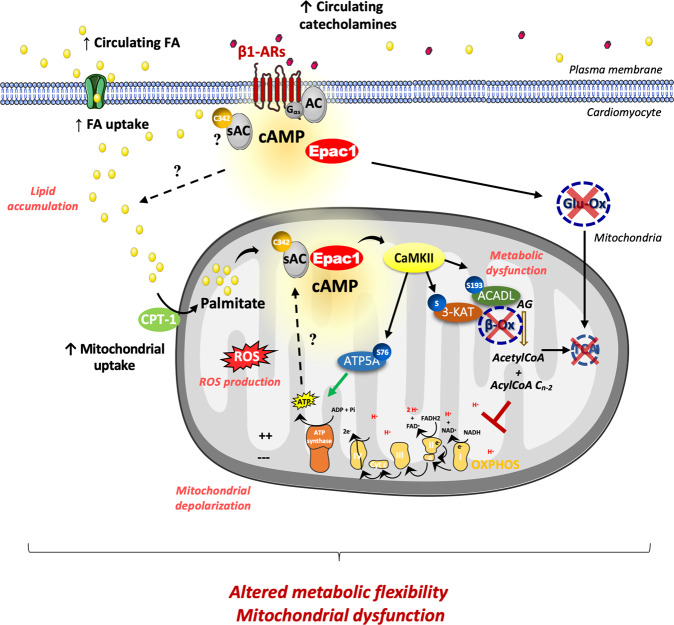
Schema illustrating a working hypothesis of Epac1 signaling during metabolic stress. In the setting of a metabolic stress, circulating levels of FA and catecholamines are increased and promote FA uptake inside the cardiomyocyte. Palmitate positively regulates Epac1 activity by soluble adenylyl cyclase (sAC) palmitoylation. Epac1 dysregulates FA metabolism (mitochondrial uptake and β-ox) via the regulation of CPT-1, β-ox enzymes, and ATP synthase by CaMKIIδ-dependent phosphorylation. Thus, Epac1 disrupts the balance between the mitochondria uptake of FA and its consumption leading to accumulation of lipid intermediates, metabolic dysfunctions (ROS production, cell death, lipid accumulation, decreased oxidative metabolism), lipotoxicity, thereby affecting metabolic flexibility and cardiac functions. Epac1 exchange protein directly activated by cAMP, FA fatty acid, cAMP cyclic AMP, CaMKII Ca^2+^/calmodulin-dependent protein kinase II, β1-ARs β1-adrenergic cardiac receptors, β-ox β-oxidation, Glu-ox glucose oxidation, CPT-1 cartinine palmitoyltransferase, ATP5A α subunit of ATP synthase, ACADL long-chain fatty acid acyl-CoA dehydrogenase, 3-KAT 3-ketoacyl-CoA thiolase, TCA tricarboxylic acid cycle, OXPHOS mitochondrial oxidative phosphorylation.

In this work, we provide novel insights into the role of cAMP-Epac1 in cardiac myocytes (Fig. 7). Specifically, we demonstrate that a saturated FA palmitate regulates Epac1 activity by stimulating cAMP production via sAC palmitoylation at a highly conserved Cys342 residue. Once activated by lipid overload, Epac1 enhanced CPT-1 activity and blocked lipid oxidation through CaMKII-dependent phosphorylation of FAO enzymes. On the contrary, Epac1 inhibition increased FAO and prevented palmitate-induced mitochondrial dysfunction. Furthermore, Epac1-CaMKII axis interacted with the ATP5A and regulated ATP synthase activity, most likely to impair mitochondrial energetics during lipid overload.

Here, we characterized an unsuspected molecular mechanism that regulated the intracellular production of cAMP. Indeed, we found that sAC was activated by palmitoylation leading to increased cAMP production and Epac1 activation. Palmitoylation is a reversible lipid modification regulating many proteins [30]. For instance, palmitoylation of the voltage-gated sodium channel, Nav1.5 or the ryanodine receptor have been shown to affect their activity and ion homeostasis in cardiomyocytes [31–33]. Here we provide evidence that sAC palmitoylation represents a novel signal transduction mechanism that is modulated by diet-dependent signals and may impact the regulation of cell fate. Aberrant sAC palmitoylation turns on an Epac1-dependent activation of CaMKII, which promotes mitochondrial dysfunction and cardiomyocyte death during chronic lipid overload. Importantly, we identified C342 as a conserved sAC palmitoylation substrate regulating sAC activity. Loss of palmitoylation at this crucial residue prevented intracellular cAMP elevation and Epac1 activation in response to palmitate. At this time, it is still unclear how palmitoylation of C342 impacts sAC activity. The complete conservation of this cysteine among sAC orthologs suggests that palmitoylation of this residue may serve as an important regulatory role in sAC activity. Of note, C342 is located in the C2 catalytic region of sAC and at the proximity of the ATP substrate binding site and HCO3^−^ regulatory binding site [26]. Therefore, this specific post-translational modification at C342 could impact sAC configuration, thus enhancing the interaction of the substrate with sAC and/or stimulating the effect of HCO_3_^−^ on sAC activity.

Our study also identified novel mechanisms that contribute to cardiomyocyte lipotoxicity. First, we characterized a novel role of cAMP in the regulation of FA mitochondrial uptake. Indeed, we found that Epac1 enhanced CPT-1 activity, a key enzyme regulating mitochondrial FA uptake, which are then degraded in the β-oxidation pathway for the production of ATP [34]. It is known that prolonged lipid overload enhanced mitochondrial ROS generation, which is followed by reduced mitochondrial respiration and ATP synthesis (decreased mitochondrial FAO) [28]. We believe that the stimulating effect of Epac1 on CPT-1 activity promotes mitochondrial FA overload and contributes to lipotoxicity due to the inability of mitochondria to entirely catabolize increased amounts of FA. Accordingly, a previous study showed that cardiac-selective inhibition of CPT-1B, the predominant CPT‐1 isoform expressed in the myocardium attenuated pathological cardiac remodeling and improved cardiac function in obese mice [35]. Yet, a selective β-blocker, metoprolol decreased CPT-1 activity and ameliorated cardiac contractility in dogs with heart failure further supporting the beneficial effect of CPT-1 inhibition in myocardial function [36]. In addition, cytosolic lipid accumulation may be due to a higher FA uptake by cardiomyocytes compared to FA mitochondrial uptake. Therefore, one cannot exclude that Epac1 may also regulate other key proteins involved in the transport of FA into the cardiomyocyte such as the plasma membrane-associated protein, FA translocase (also known as CD36), and FA transport protein 1 (FATP1).

Besides its effect on ROS production and mitochondrial potential, Epac1 may also influence cardiomyocyte death by altering the balance of mitochondrial dynamics (fusion/fission). Indeed, Yang and colleagues recently showed that Epac1-induced cardiomyoblast apoptosis in the context of hypoxia correlated with a change in the expression of key proteins involved in mitochondrial dynamics such as dynamin related protein 1 (DRP1) and mitofusin 2 (MFN2) [37]. However, in the context of lipid overload, mitochondrial morphology modification may not be a primary event in cell death. Indeed, Tsushima and collaborators reported that scavenging mitochondrial ROS prevented palmitate-induced dysregulation of mitochondrial dynamics in cardiomyocytes [28]. Therefore, the lipotoxic effect of palmitate is mainly mediated through Epac1-promoted ROS production and mitochondrial dysfunction.

Second, we characterized Epac1 as a novel regulator of oxidative metabolism in mitochondria. Pharmacological inhibition of Epac1 increased FAO in palmitate-treated cardiomyocytes under lipid overload condition. Mitochondrial Epac1 interacted with two β-oxidation enzymes, ACADL and 3-KAT to modify their phosphorylation state via CaMKII thereby decreasing FA breakdown through the β-oxidation pathway. Therefore, Epac1 activation decreases FAO degradation and together with its stimulating effect on FA uptake it facilitates intracellular accumulation of lipid intermediates thus causing mitochondrial dysfunction, ROS production and cardiomyocyte death. These findings suggest that enhancing mitochondrial FAO may prevent obesity induced cardiomyopathy. Consistently, a recent study showed that increasing mitochondrial FAO by genetic deletion of acetyl-CoA carboxylase 2 that enhanced FAO by relieving the inhibition of CPT-1 was sufficient to prevent the development of obesity induced cardiomyopathy, in part via the inhibition of mitochondrial dysfunction [38]. On the contrary, inhibiting the increase of myocardial FAO exacerbated cardiac damage in obese mice [39]. Taken together, these data suggest that Epac1 inhibition may represent a therapeutic opportunity for lipotoxic cardiomyopathy associated with obesity and diabetes.

Interestingly, we found that Epac1 pharmacological blockade not only increased ECAR, a key component of glycolysis rate but also prevented FAO-induced glucose oxidation inhibition in lipid overload condition. Hence, Epac1 inhibition could reduce glucotoxicity arising from accelerated sugar-related modifications, such as O-GlyNAcylation and advanced glycation end products (AGE) formation. Altogether, these results show that Epac1 inhibition allows a better metabolic flexibility of cardiomyocytes in the context of lipid overload.

In our study, we reported that during a metabolic stress Epac1 interacted with the α subunit of ATP synthase, ATP5A and activated ATP synthase activity via a CaMKIIδ-dependent phosphorylation of ATP5A at Ser76. Accordingly, a previous study showed that inhibition of O-GlcNAcylation on ATP5A decreased ATP synthase activity and subsequent ATP production [40] indicating that post-translational modifications of ATP5A may strongly impact the activity of this enzyme. The functional consequence of Epac1-induced ATP synthase activity has yet to be investigated but one could hypothesize that this effect of Epac1 could stimulate the production of ATP, which in turn could stimulate sAC activity to enhance Epac1 activation and its detrimental role in cardiomyocytes. Alternatively, increased activation of ATP synthase by Epac1 could accelerate mitochondrial depolarization by disrupting the proton gradient that was not restored because of deficits in reduced equivalents. In line with this hypothesis, we demonstrated here that Epac1 induced CaMKII-dependent phosphorylation of FAO enzymes such as ACADL to decrease FAO and consequently the substrates that fueled mitochondrial oxidative phosphorylation.

Interestingly, we identified a novel regulatory signaling of FAO and complex V of the respiratory chain since we provided evidence that mitochondrial CaMKII, mainly the CaMKIIδ isoform acted as a key downstream effector of Epac1 in the regulation of mitochondrial ACADL, 3-KAT, and ATP synthase. Of particular importance, we demonstrated that the regulatory effect of CaMKII on ACADL and ATP5A occurred via serine-dependent phosphorylation. This novel biological action of CaMKII in the mitochondrial compartment is in line with a previous study showing that (1) CaMKII had a mitochondrial localization, and (2) mitochondrial Epac1-CaMKIIδ axis regulated the activity of the isocitrate dehydrogenase 2, a critical mitochondrial enzyme of the tricarboxylic acid (TCA) cycle [16]. Consistent with our data, previous work indicated that CaMKII contributed to the lipotoxicity of palmitate in cardiac-derived H9C2 cells [41]. At present, further work is required to understand how Epac1 activates CaMKII in mitochondria and whether Rap-GTPase, a direct effector of Epac1, is involved in Epac1-CaMKII signalosome. Downstream effectors of CaMKII may also converge on key metabolic regulators AMP-activated protein kinase, and peroxisome proliferator-activated receptor gamma coactivator-1α, which interconnect with cAMP-Epac1 pathway [42].

In summary, we here identify a novel signaling pathway by which cAMP-Epac1 axis controls mitochondrial β-oxidation and metabolic flexibility in cardiomyocyte. We propose a model whereby, Epac1 disrupts the balance between mitochondrial FA uptake and oxidation leading to lipid accumulation, mitochondrial dysfunction, and cardiomyocyte death during lipid overload conditions. Epac1 inhibition allows a better management of substrates and energy within cardiomyocyte and therefore could have potential therapeutic implications for obesity induced cardiomyopathy.

## Materials and methods

### Animals

Mice were housed in a pathogen-free facility and all animal experiments were approved by the Animal Care and Use Committees of the University of Toulouse. Epac1-deficient mice (*Epac1*^*−/−*^*)* have been engineered in our laboratory as previously described [43].

### Cardiac lipotoxicity mice model

Mice were placed either on a high-fat diet (HFD, 60% saturated fat, 20% proteins, and 20% carbohydrate, ResearchDiet, Broogarden, Denmark) or a normocaloric diet (ND, 10% lipids, 20% proteins, and 70% carbohydrate, SSNIFF Spezialdiäten GmBH, Germany) ad libidum for 15 weeks. The animals were housed on a day/night cycle 12 h/12 h with unlimited access to food and water.

### Human heart tissues

Human samples were collected in the Cardiac Surgery Department of the Toulouse University Hospital, France, and stored in liquid nitrogen. Samples used in this study were right atrial appendage tissue, retrieved after cardio-pulmonary bypass weaning, at the site of the venous cannula withdrawal scar. Obese status was defined as a body mass index over 30 Kg/m^2^, non-obese status was defined as a body mass index under 25 Kg/m^2^. The two groups were homogeneous concerning the main cardiac pathology, the preoperative left ventricle ejection fraction, and the presence or the absence of atrial fibrillation.

### Cell culture

All procedures were performed in accordance with the Guide for the care and use of laboratory animals and the veterinary committee has been informed of the cardiac myocyte isolation protocol used. All media, sera, and antibiotics used in cell culture were purchased from Invitrogen.

#### Neonatal rat ventricular myocytes (NRVM) isolation

Neonatal Sprague-Dawley rats of 1–2 days old were euthanized by decapitation. The heart was excised and the atria were removed. Primary culture of NRVMs was subsequently performed as previously described [44]. The ventricles were pooled, and the ventricular cells were dispersed by digestion with collagenase II (Worthington) and pancreatin (Life Technologies, Inc.). The cell suspension was purified by centrifugation through a discontinuous Percoll gradient to obtain myocardial cell cultures with >95% myocytes. The cardiomyocytes were plated at a density of 5–6 × 10^4^ cells/cm^2^ in Dulbecco’s modified Eagle’s medium (DMEM, Gibco) 4,5 g/L Glucose + glutamax/medium 199 (4:1) supplemented with 10% horse serum, 5% fetal bovine serum, glutamine, and antibiotics (plating medium), and allowed to attach overnight. The cells were then washed twice with DMEM/medium 199 and further incubated in DMEM/medium 199 supplemented with glutamine and antibiotics (maintenance medium) in the presence or absence of various agents.

#### Adult murine ventricular cardiomyocytes (AMVC) isolation

After intraperitoneal injection of pentobarbital (300 mg/kg) and heparin (150 U), the hearts from WT and *Epac1*^*−/−*^ were rapidly isolated and placed in ice-cold Tyrode calcium free buffer (130 mM NaCl, 5.4 mM KCl, 1.4 mM MgCl_2_, 0.4 mM NaH_2_PO_4_, 4.2 mM HEPES, 10 mM glucose, 20 mM taurine, and 10 mM creatine monohydrate, pH 7.2). The heart was quickly excised, and the aorta was cannulated for retrograde perfusion in a Langendorff apparatus at a constant flow rate of 3 mL/min at 37 °C. Primary culture of AMVCs was subsequently performed as previously described [16]. The heart was perfused for 6–8 min with Tyrode buffer, followed by Tyrode buffer containing 12.5 µM CaCl_2_ and 0.5 mg/mL Liberase™ medium Thermolysin (Roche Diagnostics, Meylan, France) for 9 min. Once the enzymatic digestion of the heart completed, the left ventricle was dissected, minced with scissors and homogenized with a pipette in Tyrode buffer containing 12.5 µM CaCl_2_ and 5% bovine serum albumin (Sigma–Aldrich, France) to stop the enzymatic reaction. Dispersed myocytes were then filtered through a 100 µm mesh and allowed to sediment by gravity for 10 min. The supernatant was removed and centrifuged for 1 min at 10 × *g*. The pellet was resuspended in Tyrode buffer containing 12.5 µM CaCl_2_. The calcium concentration was increased gradually from 12.5 µM to 1 mM in five steps over ~20 min. Freshly isolated cardiomyocytes were plated for 2 h on 35 mm laminin (10 μg/mL)-coated tissue culture dishes in M199 complete medium (M199 medium with added 100 IU/mL penicillin, 2 mM L-carnitine, 5 mM creatine, and 5 mM taurine). After this period of attachment, the medium was changed and cells were incubated overnight at 37 °C in a humidified atmosphere of 5% CO_2_ and air. The culture protocol yielded an average of 80% rodshaped myocytes at a plating density of 50 cells/mm^2^ that were viable at pH 7.2 for 48 h. Experiments were performed the day following isolation and culture.

### Plasmid constructs and transfection

The mutant form of Epac1 deleted for its putative mitochondrial-targeting sequence (Epac1^∆2–37^) was producted by mutagenesis in a previous study [16]. The plasmid encoding the wild-type mitochondrial soluble adenylate cyclase (sAC^WT^) was provided by Dr. Gergő Szanda. The expression vector (pCMV3) encoding wild-type forms of ATP5A and ACADL enzymes were purchased from SinoBiological Inc. The mutant form of sAC (sAC^C342A^), ACADL^S193A^, and ATP5A^S76A^ were producted by mutagenesis (QuickChange Site-Directed Mutagenesis Kit, Agilent Technologies). Primary neonatal rat cardiomyocytes were transfected using Lipofectamine 2000 (Invitrogen) according to the manufacturer’s instructions.

### Reagents and antibodies

The highly membrane-permeant Epac1 agonist 8-(4-Chlorophenylthio)-2’-O-methyladenosine-3’, 5’-cyclic monophosphate, acetoxymethyl ester (8-CPT-AM) and its respective control (AM) were purchased from BioLog (Bremen, Germany). The Epac1 inhibitor CE3F4 was synthesized according to the methods published previously [23]. The following commercially available inhibitors were used: sAC inhibitor, KH7 (Cayman Chemical, Bertin Pharma, Montigny-le-Bretonneux, France); CaMKII inhibitor, KN-93 (Sigma–Aldrich, France); Cartinine palmitoyltranferase CPT-1 inhibitor, etomoxir (Sigma–Aldrich, France); ATP synthase inhibitor, oligomycin (Sigma–Aldrich, France); mitochondrial respiratory chain complex I inhibitor, rotenone (Sigma–Aldrich, France); and mitochondrial respiratory chain complex III inhibitor, antimycin A (Sigma–Aldrich, France). Carbonyl cyanide 4- (trifluoromethoxy)phenylhydrazone (FCCP), a protonophore decoupling the respiratory chain, D-Glucose and 2-deoxyglucose, BSA solution, sodium palmiate and BSA-Oleic acid (oleate) were purchased from Sigma–Aldrich. BSA-palmitic acid (palmitate) was produced and supplied by Dr. Cedric Moro (I2MC, France). Primary antibodies for immunoblot were obtained from the following sources: Epac1 (#4155), Rap1 (#4938), Myc-Tag (#2276) (all at 1/1000) and GAPDH (#2118, at 1/5000) from Cell Signaling (Ozyme, Montigny-le-Bretonneux, France). ACADL, ATP5A, and 3-KAT were immunoprecipitated using an antibody against Myc-Tag (#2276, Cell Signaling). Primary antibodies against phosphoserine (#ab9332), phosphotyrosine (#ab10321), phosphothreonine (#ab9337), CaMKIIδ (#ab181052), ADCY10 (#ab82854), ACADL (#ab128566), ATP5A (#ab14748), and 3-KAT (#ab230667) were from Abcam (all at 1/1000) and tubulin (#T8203, at 1/5000) from Sigma–Aldrich. Unrelevant antibody for immunoprecipitation control (IgG1 Isotype Control, #5415) was from CellSignaling. OXPHOS antibody cocktail was purchased from Mitoscience. All media, sera, and antibiotics used in cell culture were purchased from Invitrogen.

### Seahorse Agilent XFe24 experiments

#### Mitochondrial respiration

Oxygen consumption rate (OCR) was measured with a Seahorse XFe24 analyzer (Agilent). ARVMs were seeded in 24-well Seahorse assay plates in complete medium and, after 2 h of cell attachment, the medium was replaced with XF basal medium supplemented with 10 mM glucose, 4 mM glutamine, and 1 mM sodium pyruvate (pH 7.4). Plates were incubated for an additional 1 h at 37 °C in a CO_2_-free incubator. The hydrated wells of the sensor cartridge were then loaded with 1 μM oligomycin (port A), 1 μM carbonyl cyanide 4- (trifluoromethoxy) phenylhydrazone (FCCP) (port B) and 1 μM antimycin A + 1 μM rotenone (port C). The data were analyzed using the Seahorse Wave software.

#### FA oxidation

Oxidation of FA in mitochondria was measured with the Seahorse Agilent technology. After cell adhesion, complete medium was replaced with XF basal medium supplemented with 4 mM glutamine and 0.5 mM carnitine (for CPT-1 function) (pH 7.4). Plates were incubated for an additional 1 h at 37 °C in a CO_2_-free incubator. The hydrated wells of the sensor cartridge were then loaded with 2 μM oligomycin (port A), 2 μM FCCP (port B) and 4 μM antimycin A + 2 μM rotenone (port C). Fifteen minutes before the start of the measurement, the cells were treated with 40 μM of etomoxir. At the time of starting the measurement, 500 μM palmitate is added. The data were analyzed using the Seahorse Wave software.

#### Glycolysis function

Extracellular acidification rate (ECAR) was determined with a Seahorse XFe24 analyzer (Agilent) that allows measuring mitochondrial glucose oxidation. After cell adhesion, plating medium was replaced with a XF basal medium supplemented with 4 mM L-glutamine (pH 7.4). Plates were incubated for an additional 1 h at 37 °C in a CO_2_-free incubator. The hydrated wells of the sensor cartridge were then loaded with 25 mM D-glucose (port A), 2 μM oligomycin (port B), and 50 mM 2-deoxyglucose (port C). Data were analyzed using the Seahorse Wave software.

### Mitochondrial ROS measurement

Cells were incubated with 5 µM of MitoSOX™ Red probe (Thermo Scientific) for 10 min to 37 °C, protected from light and then washed with PBS. Red fluorescence was measured at 580 nm after excitation of the probe at 510 nm using a fluorimeter.

### SiRNA experiments

Rat CaMKIIδ-specific siRNA and control siRNA (Horizon Discovery) were resuspended at 20 µM in distilled water. One day after plating, primary cardiomyocytes were transfected were transfected with DharmaFECT® Duo (Thermo Scientific) using 100 nM of siRNA per well.

### Assessment of mitochondrial membrane potential (ΔΨm)

ΔΨm was monitored using the fluorescence dye 5′,6,6′-tetrachloro-1,1′,3,3′-tetraethyl-benzamidazolocarbocyanin iodide (JC-1, Enzo Life Sciences, Villeurbanne, France). Cells were incubated with 5 µM JC-1 in M199 complete medium at 37 °C for 15 min and then washed with PBS. Excitation of the dye was performed at 496 nm and emission signal ratio (590/530) was normalized to that measured under VEHICLE condition.

### Rap activation assay

Rap1 pull-down experiments were performed using the Rap1 binding domain of Ral-GDS fused to GST as described previously [23]. Cells were starved for 1 h before stimulation in serum-free MEM containing penicillin-streptomycin (1%). After transfection, cells were lysed in radioimmune precipitation assay buffer (50 mM Tris-HCl pH = 7.5, 500 mM NaCl, 20 mM MgCl_2_, 0.5% deoxycholic acid, 0.1% SDS, 1% Triton X-100, 1 mM PMSF, protease, and phosphatase inhibitors), and 500 μg of protein was incubated with Ral-GDS coupled to glutathione-Sepharose beads (Amersham Biosciences) for 1 h at 4 °C. The beads were then washed three times in washing buffer (50 mM Tris-HCl pH = 7.5, 150 mM NaCl, 20 mM MgCl_2_, 1% Triton X-100, 0.1 mM PMSF, protease, and phosphatase inhibitors) and then resuspended and heated for 10 min at 95 °C in Laemmli to perform immunoblots.

### Acyl-biotin exchange method (ABE)

ABE experiments experiments were performed as previously described [25]. Briefly, cell lysates were incubated overnight at 4 °C with N-ethylmaleimide (Thermo Scientific) to block free cysteine and to ensure complete cysteine alkylation. The next day, the insoluble component was removed after centrifugation at 13,500 × *g* for 5 min. The proteins were precipitated with chloroform/methanol to remove excess chemicals. After the last centrifugation, proteins were dissolved in 1 mL of PBS containing 1% SDS. The soluble protein was divided into two equal groups. The first group was resuspended in a solution containing 0.7 M hydroxylamine NH_2_OH, 1 mM biotin, 0.2% Triton X-100 supplemented with protease inhibitors. The second one was treated with 200 mM Tris, 1 mM biotin, 0.2% Triton X-100 supplemented with antiproteases. Hydroxylamine (HAM, Sigma–Aldrich) is a powerful reducing agent that cleaves the palmitate from cysteines to allow biotinylation. Sites blocked by N-ethylmaleimide (Thermo Scientific) were not cleaved. The lysates were also treated simultaneously with the biotin-HPDP reagent (Thermo Scientific). The remaining chemicals were removed using chloroform/methanol precipitation and resolubilized in PBS containing 1% Triton X-100 and 0.2% SDS. The biotinylated proteins were captured by streptavidin-Sepharose beads (GE-Healthcare) for 2 h at room temperature. Then, beads were washed four times with PBS containing 1% Triton X-100; 0.2% SDS and then resuspended and heated for 10 min at 95 °C in Laemmli to perform immunoblots.

### Immunoprecipitation

Cardiomyocytes were mixed overnight at 4 °C with 5 µg of appropriated antibody in buffer containing 20 mM Tris, pH 7.5, 150 mM NaCl, 1 mM EDTA, 1% Triton X-100, protease, and phosphatase cocktail inhibitors (Roche). Purification steps were performed with protein A/G agarose according to the manufacturer’s instructions (SantaCruz).

### Western blot

Once denatured, the samples were deposited and separated by SDS-PAGE electrophoresis (20 min at 80 V then 2 h at 130 V at room temperature). The proteins were then transferred to a PVDF membrane, previously activated in 100% ethanol for 5 min, using the Trans-blot transfer apparatus (20 V, 1 A) for 30 min. The membrane was then blocked in a solution of TBS-Tween 20-BSA 3% minimum 1 h at room temperature. After incubation with the primary antibody overnight at 4 °C, a secondary antibody or Protein G-HRP (dilution 1:5000, Biorad) was added for 90 min at room temperature. Only the membranes having fixed samples resulting from immunoprecipitation experiments were incubated with Protein G-HRP in order to overcome the labeling of heavy and light chains of the denatured antibody. After washing with TBS-Tween, membranes were revealed by chemiluminescence using GE-Healthcare Amersham ECL Kit and readed with BioradChemDocTM XRS+. Quantification was performed using the ImageLab software and normalized to a household protein (GAPDH (Cell Signaling) or Tubulin (Sigma–Aldrich)).

### Capillary western blot

Epac1 protein quantification was achieved by capillary western blot analysis using the ProteinSimple Jess system with 12–230 kDa Jess separation module capillary cartridges (ProteinSimple, SantaClara, CA, USA). A mouse monoclonal antibody specific for Epac1 (Cell Signaling) was used (1:50). Anti-mouse detection module for Jess (ProteinSimple) kit included Luminol-S, peroxide, antibody diluents, streptavidin coupled horseradish peroxidise, anti-mouse secondary antibody, and a protein normalization reagent. Sample proteins (5 ng per each condition) were allowed to separate via the capillary technology and were analysed based on chemiluminescence, which was transformed into digital images depicting bands as observed in western blots. The abundance of Epac1 and total proteins were determined using Compass software (ProteinSimple). The normalized data are expressed as Epac1 signal intensity over total proteins signal intensity.

### Lactate dehydrogenase (LDH) release

LDH release in the cell culture medium was measured according to the manufacturer’s instructions (LDH-Cytotoxicity Assay Kit II, Abcam).

### MTT assay

Cells were plated in 100 μL in 96-well flat bottom plates and then exposed to tested agents. At the end of treatment, 10 μL of 5 mg/mL MTT (3-[4,5-dimethylthiazol-2-yl]-2,5-diphenyltetrazolium bromide; thiazolyl blue, Sigma–Aldrich) solution in PBS were added to each well for 3 h. After removal of the medium, 50 μL of DMSO were added to each well to dissolve the formazan crystals. The absorbance at 550 nm was determined using a plate reader (TECAN infinite F500). Triplicate wells were assayed for each condition.

### cAMP quantification

Analysis of the total cellular cAMP content was performed using the cAMP direct Enzyme Immunoassay Kit (Enzo Life Sciences). Briefly, supernatants were removed and cells were treated with 0.1 M HCl for 10 min at RT and were centrifuged ≥600 × *g* to pellet the cellular debris. The supernatant was either assayed immediately or stored frozen (−20 °C) for later analysis according to the manufacturer’s instructions.

### Palmitate oxidation

Cardiomyocytes were incubated for 3 h with [1-^14^C]palmitate (1 μCi/ml; Perkinelmer, Boston, MA) and nonlabeled (cold) palmitate at a final concentration of 100 µM. Palmitate was coupled to a FA–free BSA in a molar ratio of 5:1. Following incubation, ^14^CO_2_ and ^14^C-ASM (acid soluble metabolites) were measured as previously described [45]. Briefly, assayed medium was transferred into a custom-made Teflon 48-well trapping plate. The plate was clamped and sealed, and perchloric acid was injected through the perforations in the lid into the medium, which drives CO_2_ through the tunnel into an adjacent well, where it is trapped in 1 N NaOH. Following trapping, the media was spun twice and ^14^C-ASM measured by scintillation counting. Aliquots of NaOH and medium were transferred into scintillation vials, and radioactivity was measured on a multipurpose scintillation counter (LS 6500; Beckman Coulter). All assays were performed in triplicates, and data normalized to cell protein content.

### Carnitine palmitoyltranferase 1 (CPT-1) activity

Cells were lysed in a buffer containing 20 mM Tris pH 7.5, 150 mM NaCl, 1 mM EDTA, Triton 1%, protease inhibitors, and phosphatases. CPT-1 activity was determined by spectrophotometry by measuring the amount of the reduced form of coenzyme A (CoA-SH) released using the Ellman reagent (5,5’-dithio-bis (2-nitrobenzoic acid (DTNB), Sigma–Aldrich) as previously described [46]. In the presence of DTNB, CoA-SH colors the medium in yellow. Briefly, cells were incubated with a reaction buffer (20 mM HEPES pH 7.4, 220 mM sucrose, 40 mM KCl, 1 mM EGTA, 0.1 mM DTNB) and the absorbance was measured for 90 min (every 30 s) at 412 nm after adding 40 μM palmitoyl-CoA and 5 mM L-carnitine. CPT-1 activity was defined in nmol CoA-SH released/min/mg protein and expressed as a percentage of the control condition.

### Assessment of ATP synthase activity

ATP synthase activity was determined with the ATP Synthase Enzyme Activity Microplate Assay Kit (Abcam) in accordance with the manufacturer’s instructions. Activities were measured in pM/mg of protein and expressed as a percentage of the control condition.

### Blue native PAGE (BN-PAGE) analysis of mitochondrial supercomplexes (SCs) and in-gel activity

BN-PAGE was performed using the Native PAGE TM system (Invitrogen). Briefly, 100 µg of isolated cardiac mitochondria were solubilized by digitonin (8 g/g protein) for 20 min on ice, then centrifuged at 20,000 × *g* at 4 °C for 10 min. After centrifugation, coomassie blue G-250 (Invitrogen) was added to the supernatant to obtain a dye/detergent concentration ratio of 1/4 and then the protein was loaded into a 4–12% nondenaturing polyacrylamide gel (Invitrogen). After electrophoresis, the complexes and SCs can be visualized by a simple Coomassie staining and destaining method. For in-gel activity analysis, 35 mM Tris, 270 mM glycine, 14 mM MgSO_4_, 10 mM ATP, and 0.2% Pb(NO_3_)_2_ in water were added to the 4–12% nondenaturing polyacrylamide gel after electrophoresis and incubated for overnight at RT. The reaction was stopped with 50% of methanol. Appearance of transparent/silver bands is indicative of complex V activity. Since the bands are transparent, the gel should be inverted (to black background) after scanning to visualize the activity clearly. The activity was determined by analyzing the inverted black bands using Image J.

### Lipid accumulation

Red oil is a Sudan-type fat-soluble dye that attaches specifically to neutral lipids (triglycerides). After fixation with 4% paraformaldehyde for 10 min, cardiomyocytes were incubated with Oil Red O working solution (60% of 0.5% red oil (0.94 g dissolved in 150 mL isopropyl alcohol) diluted in 40% of distilled water), for 20 min at RT. After washing with PBS, slides were mounted with Vectashield in an aqueous medium and then cells were observed under a phase contrast fluorescence microscope.

### Quantification and statistical analysis

Data are presented as mean or fold increase ±SEM. No statistical analysis was used to predetermine sample size. Estimates were made based on our previous experience, experimental approach, availability of the samples. Prespecified exclusion criteria in cell experiments is absence of response in the palmitate only condition for expected effects (cell death, ROS production, mitochondrial membrane potential). No exclusion criteria were applied in the others experiments. All experiments were performed with at least three independent biological replicates (exact number is reported in figures’ legend). Cages housing mice were randomly assigned to experimental or control diet. Investigators were blinded to the group of the individual animals or patients during the outcome assessments. Analyses were performed using Prism 7 (GraphPad Software). Data distribution within groups/conditions were validated by the Shapiro-Wilk normality test. Multiple comparisons were performed with one-way or two-way analysis of variance (ANOVA) as appropriate followed by post-hoc test with Bonferroni correction for multiple comparison, if normality test was not passed the nonparametric Kruskal–Wallis test was performed. Parametric unpaired two-tailed *t*-test was used for comparisons of two groups, if normality test was not passed the nonparametric Mann–Whitney test was used. Two-tailed *p*-value were used. Asterisk (*) indicates *p* < 0.05; **p < 0.01; ***p < 0.001; ****p < 0.0001; ns, not significant. Differences in means were considered statistically significant at *p* < 0.05.

## Supplementary information

Figure S1

Figure S2

Figure S3

Figure S4

Figure S5

Figure S6

Figure S7

Figure S8
