# Supplementary figures and images for: Cyclic AMP-binding protein Epac1 acts as a metabolic sensor to promote cardiomyocyte lipotoxicity
Source: Cell Death Dis. 2021 Sep 1;12(9):824. doi: 10.1038/s41419-021-04113-9 (PMC8410846; doi:10.1038/s41419-021-04113-9)

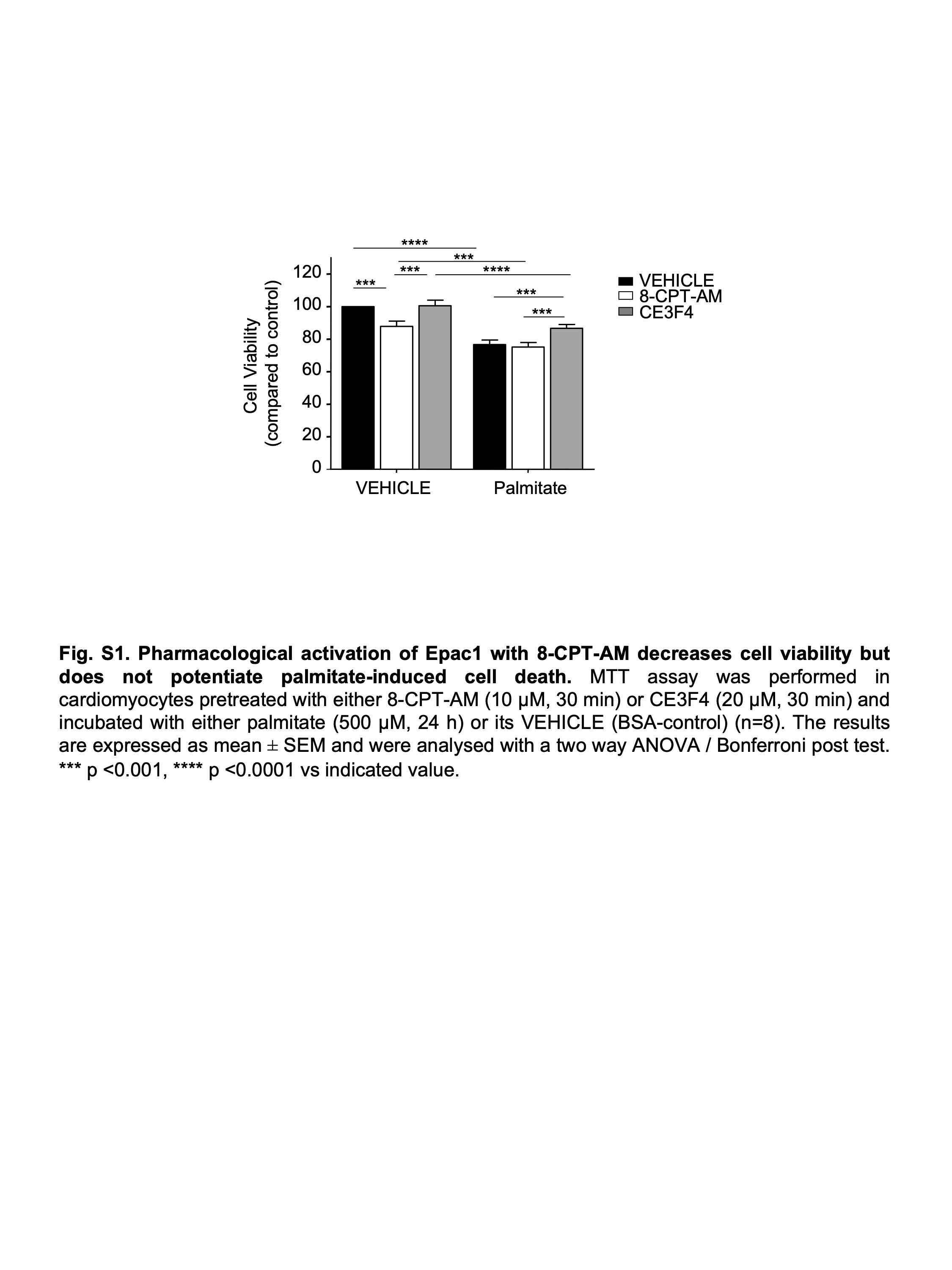

Supplement: Supplementary file 1 — Figure S1 [file 41419_2021_4113_MOESM1_ESM.tif]

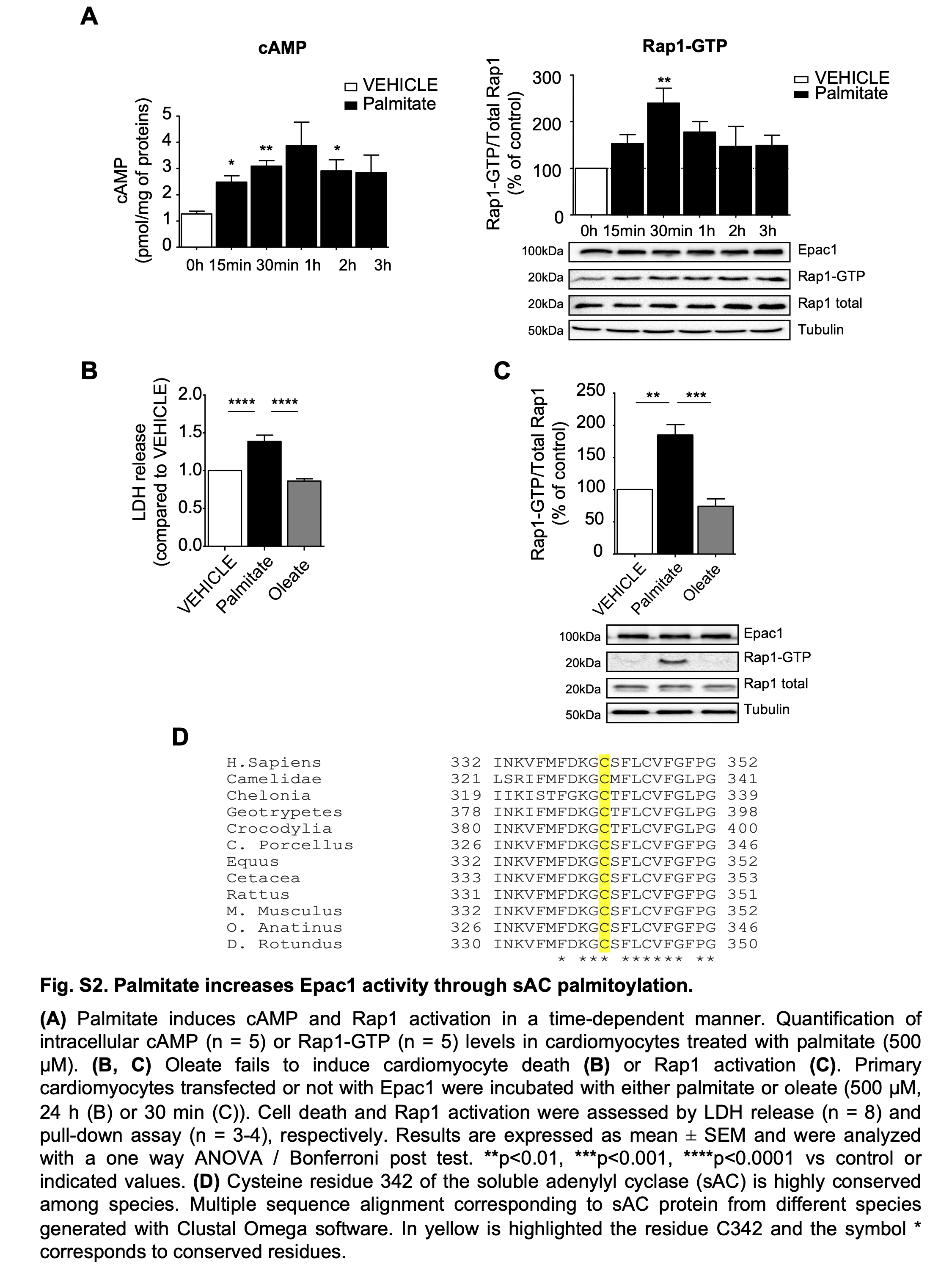

Supplement: Supplementary file 2 — Figure S2 [file 41419_2021_4113_MOESM2_ESM.tif]

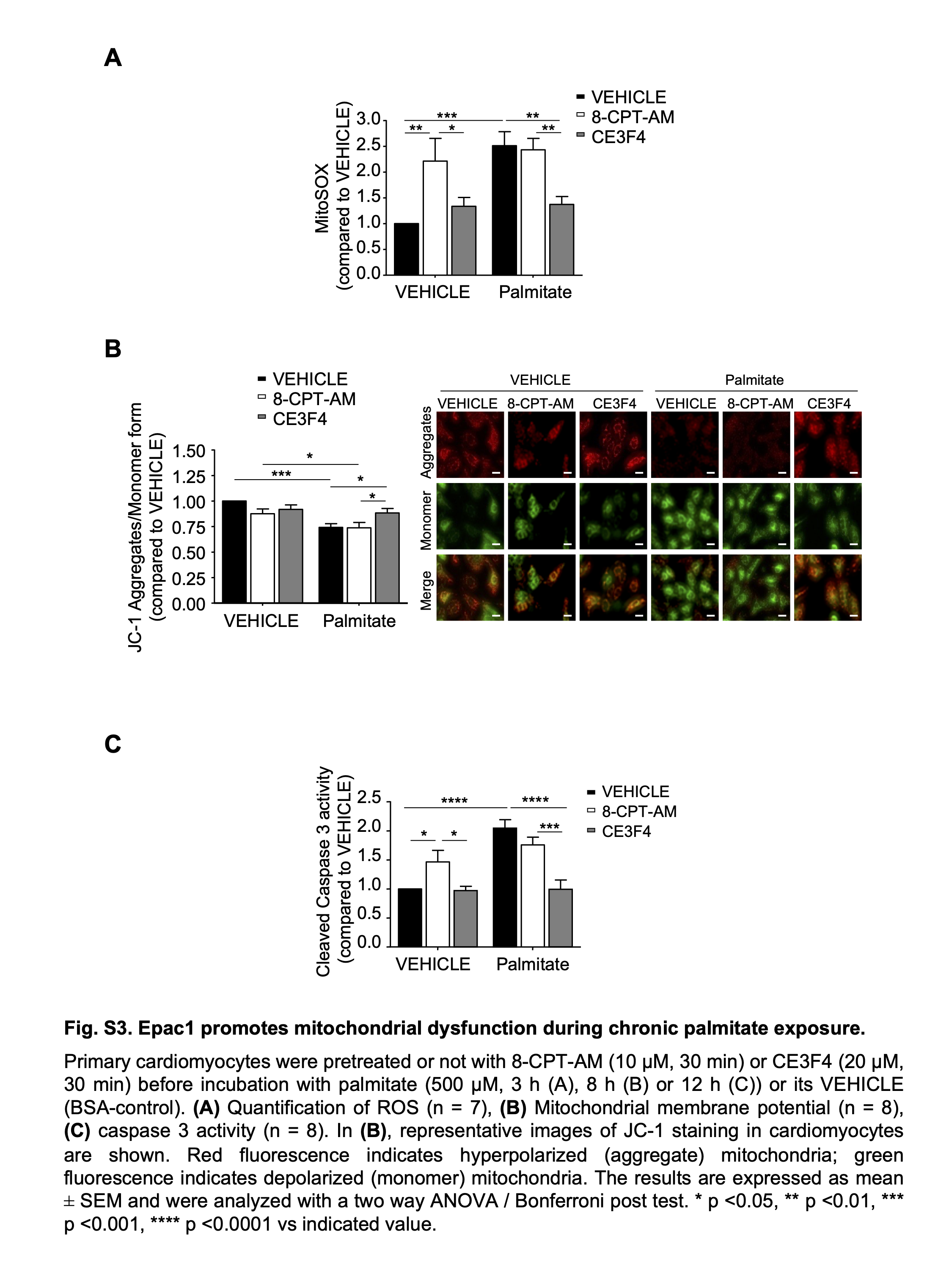

Supplement: Supplementary file 3 — Figure S3 [file 41419_2021_4113_MOESM3_ESM.tif]

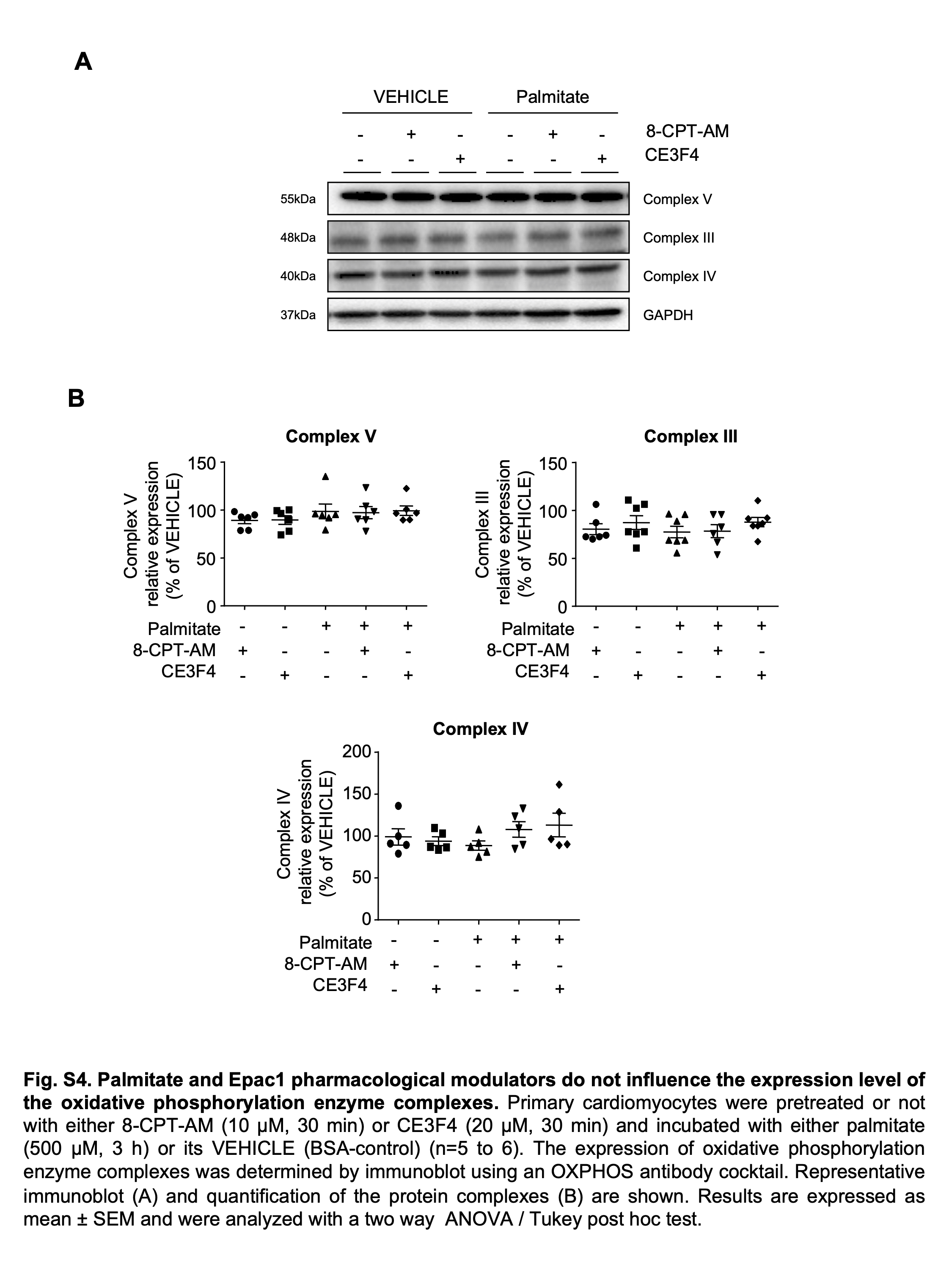

Supplement: Supplementary file 4 — Figure S4 [file 41419_2021_4113_MOESM4_ESM.tif]

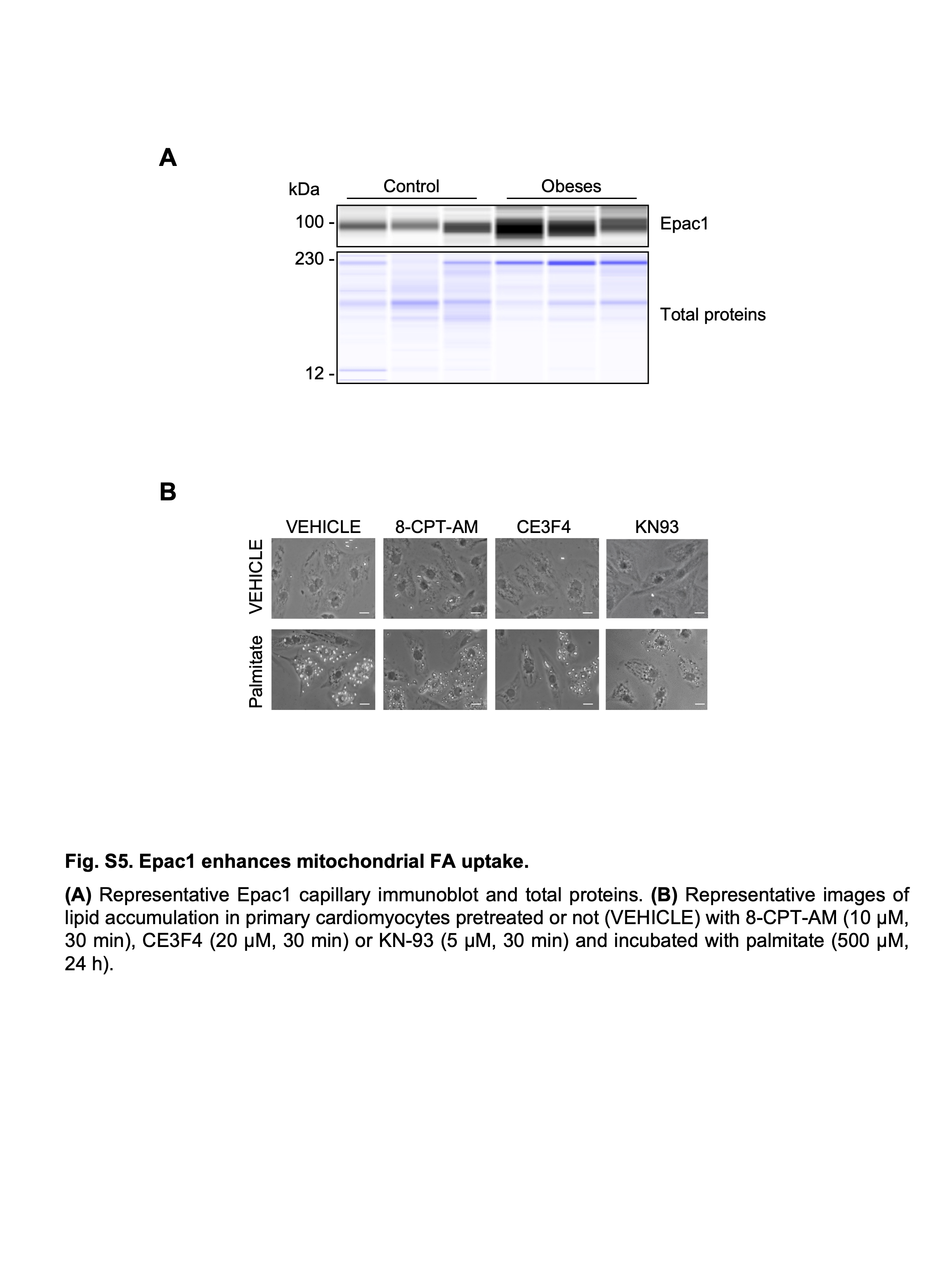

Supplement: Supplementary file 5 — Figure S5 [file 41419_2021_4113_MOESM5_ESM.tif]

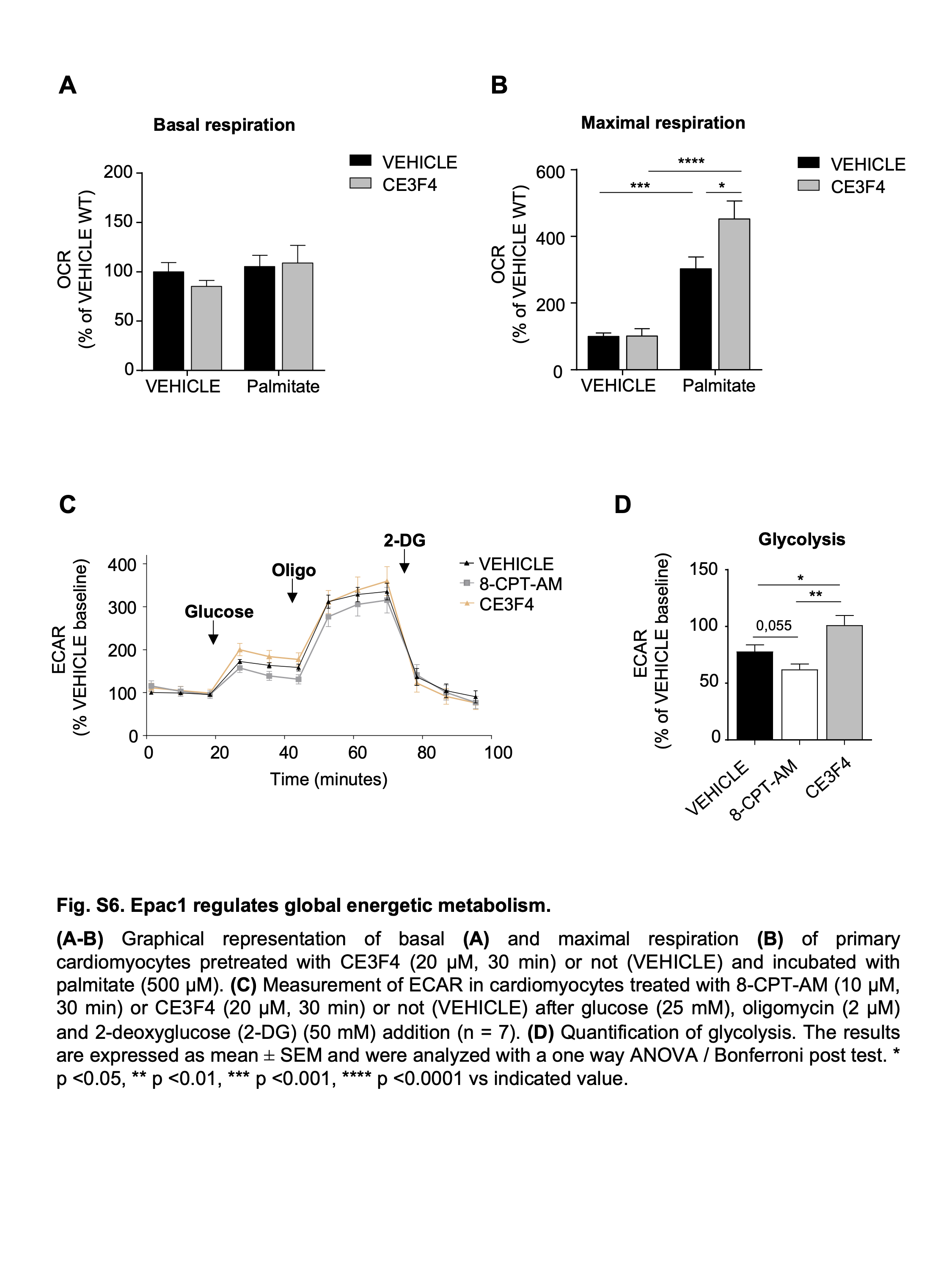

Supplement: Supplementary file 6 — Figure S6 [file 41419_2021_4113_MOESM6_ESM.tif]

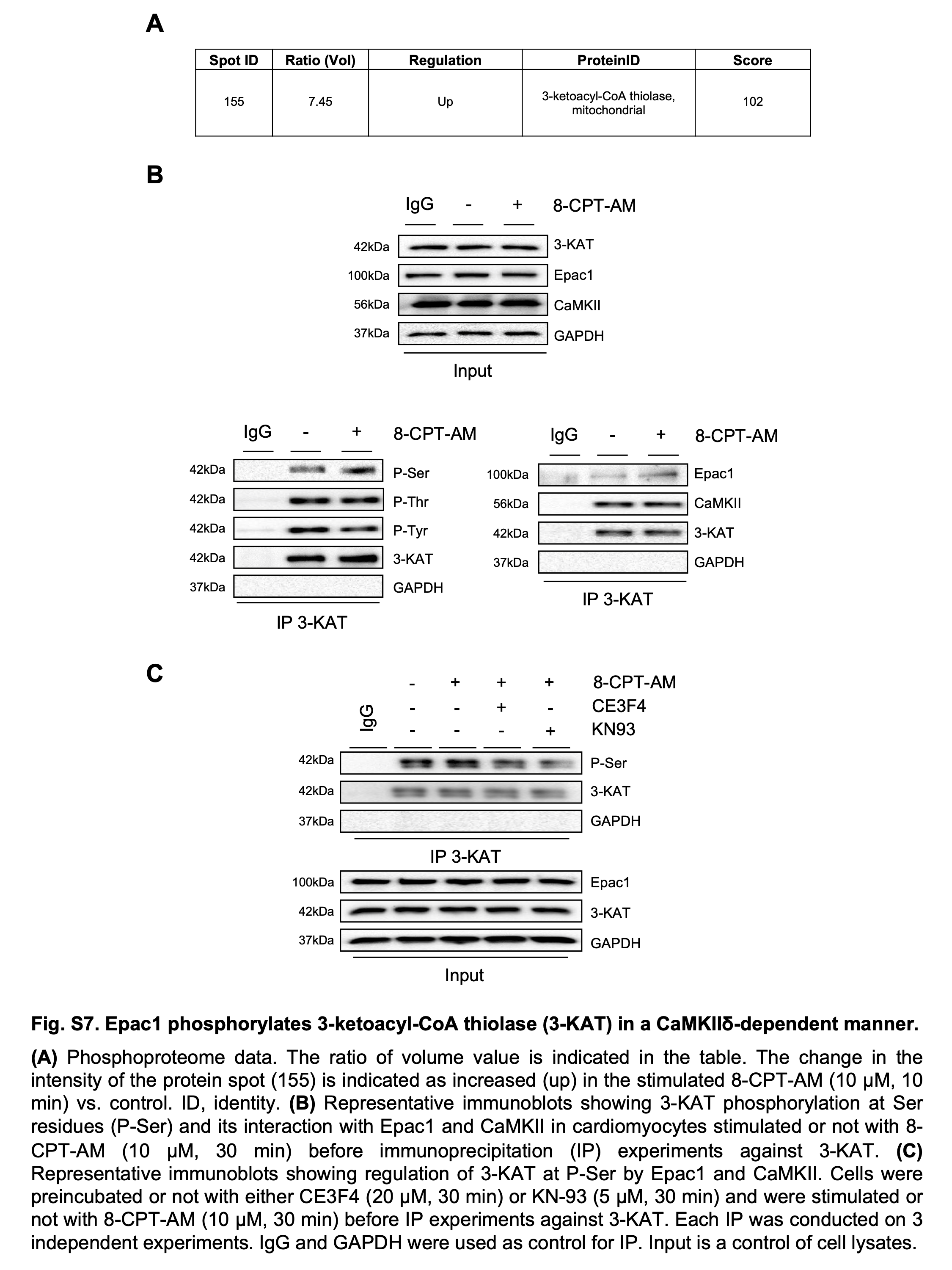

Supplement: Supplementary file 7 — Figure S7 [file 41419_2021_4113_MOESM7_ESM.tif]

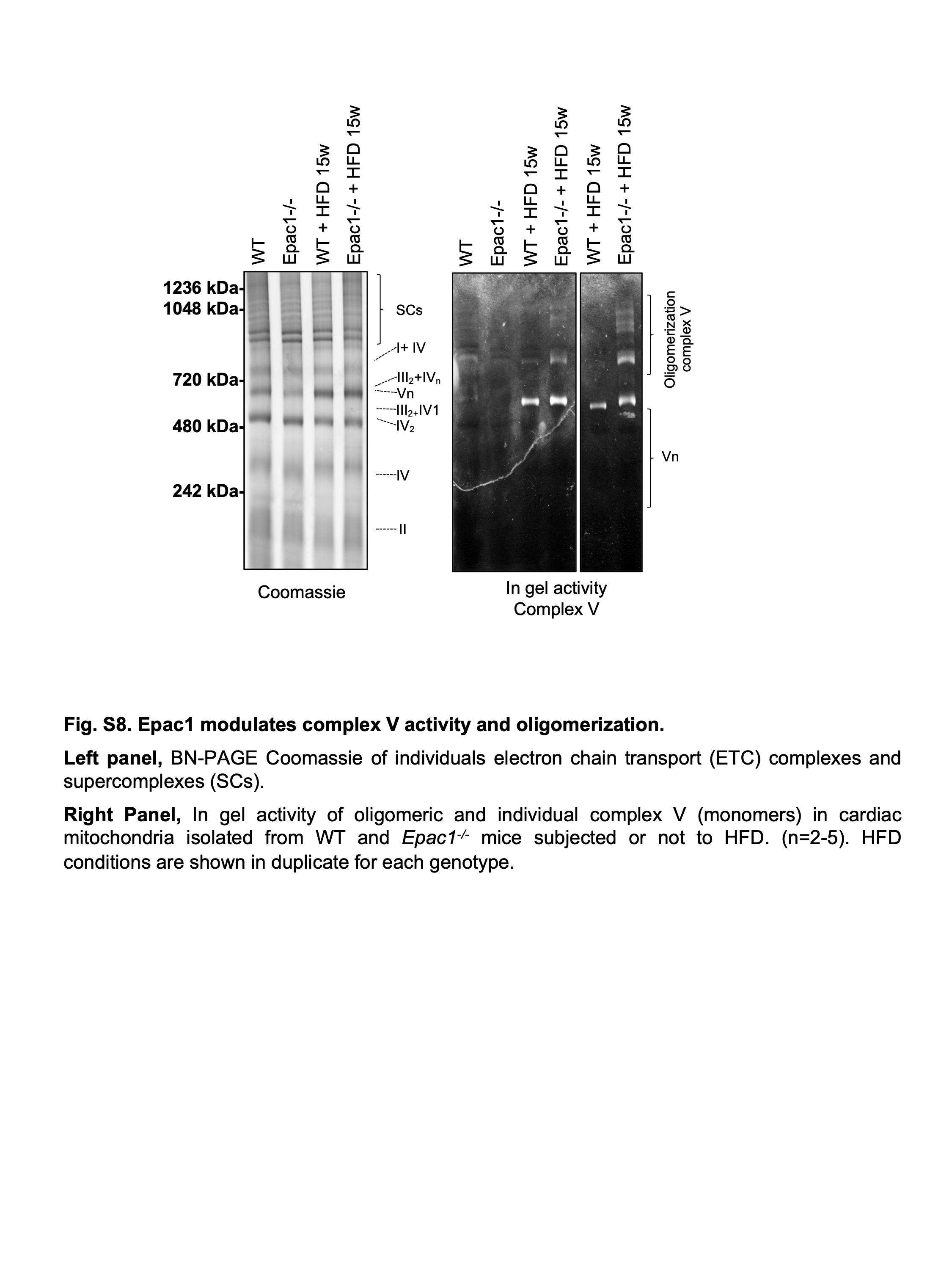

Supplement: Supplementary file 8 — Figure S8 [file 41419_2021_4113_MOESM8_ESM.tif]
